# Co-translational capturing of nascent ribosomal proteins by their dedicated chaperones

**DOI:** 10.1038/ncomms8494

**Published:** 2015-06-26

**Authors:** Patrick Pausch, Ujjwala Singh, Yasar Luqman Ahmed, Benjamin Pillet, Guillaume Murat, Florian Altegoer, Gunter Stier, Matthias Thoms, Ed Hurt, Irmgard Sinning, Gert Bange, Dieter Kressler

**Affiliations:** 1 LOEWE Center for Synthetic Microbiology (SYNMIKRO) and Department of Chemistry, Philipps-University Marburg, Hans-Meerwein-Straße, Marburg D-35043, Germany; 2 Unit of Biochemistry, Department of Biology, University of Fribourg, Chemin du Musée 10, Fribourg CH-1700, Switzerland; 3 Heidelberg University Biochemistry Center (BZH), Im Neuenheimer Feld 328, Heidelberg D-61920, Germany

## Abstract

Exponentially growing yeast cells produce every minute >160,000 ribosomal proteins. Owing to their difficult physicochemical properties, the synthesis of assembly-competent ribosomal proteins represents a major challenge. Recent evidence highlights that dedicated chaperone proteins recognize the N-terminal regions of ribosomal proteins and promote their soluble expression and delivery to the assembly site. Here we explore the intuitive possibility that ribosomal proteins are captured by dedicated chaperones in a co-translational manner. Affinity purification of four chaperones (Rrb1, Syo1, Sqt1 and Yar1) selectively enriched the mRNAs encoding their specific ribosomal protein clients (Rpl3, Rpl5, Rpl10 and Rps3). X-ray crystallography reveals how the N-terminal, rRNA-binding residues of Rpl10 are shielded by Sqt1's WD-repeat β-propeller, providing mechanistic insight into the incorporation of Rpl10 into pre-60S subunits. Co-translational capturing of nascent ribosomal proteins by dedicated chaperones constitutes an elegant mechanism to prevent unspecific interactions and aggregation of ribosomal proteins on their road to incorporation.

## Abstract (web summary)

The synthesis of ribosomes requires the orderly assembly of many proteins and large RNA molecules, a process that involves several assembly factors. Here the authors show that dedicated chaperones capture the N termini of specific nascent ribosomal proteins to promote folding and assembly into maturing ribosomes.

## Graphical abstract

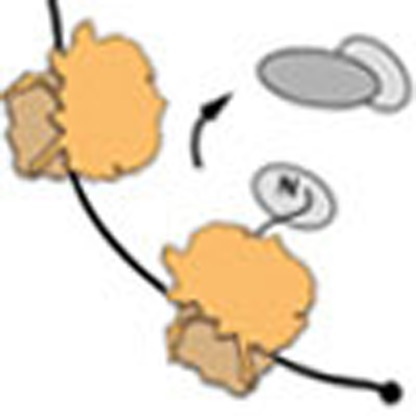

Ribosomes are the macromolecular nanomachines that translate the genetic information contained within mRNAs into all cellular proteins. While the mechanism of peptide bond formation is universally conserved, the composition and architecture of both the small 40S (SSU) and large 60S (LSU) eukaryotic ribosomal subunits (r-subunits) are significantly more complex than the one of their prokaryotic counterparts^1^,^2^,^3^,^4^. In the yeast *Saccharomyces cerevisiae*, the 40S r-subunit is composed of 33 ribosomal proteins and the 18S ribosomal RNA (rRNA), and the 60S r-subunit contains 46 ribosomal proteins and the 25S, 5.8S and 5S rRNA^3^. The synthesis of ribosomal proteins and rRNAs occupies a substantial part of transcriptional and translational capacity of the cell^5^. An exponentially growing yeast cell produces ∼2,000 ribosomes per minute, and must therefore synthesize at least 160,000 new ribosomal proteins per minute^5^. This also imposes, due to the physical separation of the nuclear and cytoplasmic compartments, a special burden on the nucleocytoplasmic transport machinery^5^,^6^. Given the high abundance and difficult physicochemical properties of ribosomal proteins^1^,^5^,^7^, their correct folding and fail-safe targeting to the assembly site relies on general, as well as highly specialized, chaperone and transport systems^8^,^9^,^10^,^11^,^12^,^13^,^14^ (see below).

The biogenesis of ribosomes is conserved among eukaryotes^15^,^16^,^17^, but most of our current knowledge concerning this intricate process, which relies on a multitude (>200) of transiently acting biogenesis factors^16^,^18^,^19^, comes from studies with the yeast *S. cerevisiae*. In yeast, ribosome biogenesis starts in the nucleolus with the recruitment of SSU ribosomal proteins and early-associating biogenesis factors to the nascent primary rDNA transcript, termed 35S pre-rRNA, thus leading to the formation of the first pre-ribosomal particle (90S/SSU processome)^18^,^20^,^21^. Endonucleolytic cleavage at processing site A_2_ then yields the pre-40S (43S) and early pre-60S particles (66S; ref. 18). Pre-40S subunits contain only relatively few biogenesis factors and are rapidly exported through the nuclear pore complex to the cytoplasm^22^, where the final maturation steps take place^23^,^24^,^25^,^26^,^27^. Maturation of nuclear pre-60S particles involves a series of sequential steps that lead to a reduction of complexity and the acquisition of export competence^16^,^18^. Export of pre-60S subunits is mainly mediated, via recognition of the export adaptor Nmd3, by the exportin Crm1 (refs 18, 28). Upon arrival in the cytoplasm, the remaining biogenesis factors are released and the last ribosomal proteins, including Rpl10 (uL16 according to the newly proposed nomenclature^29^), get incorporated, thus enabling subunit joining and engagement of 80S ribosomes in translation^30^,^31^.

Most ribosomal proteins, besides containing in many cases a globular domain, are made up of long extensions that mostly penetrate into the interior and stabilize the tertiary structure of rRNA^1^. These extensions, which are often devoid of secondary structure elements, are especially rich in lysine and arginine residues, and thus may cause aggregation of ribosomal proteins, when these are not incorporated into their cognate rRNA environment, in the presence of nonspecific polyanions^32^. Moreover, proper folding of ribosomal proteins especially depends on the integrity of two functionally collaborating ribosome-associated chaperone systems^10^,^12^. Upon release from cytoplasmic ribosomes, most newly synthesized ribosomal proteins need to be transported by importins through the nuclear pore complex in order to reach their assembly site in the nucleus^8^. It could be shown that importins not only act as nuclear import receptors but also fulfil a role as chaperones for proteins with exposed basic domains, such as ribosomal proteins^32^. However, recent evidence revealed that certain ribosomal proteins interact with specific binding partners, also referred to as chaperones^9^,^11^. By likely acting, in contrast to the classical folding chaperones, as holding chaperones^33^, these binding partners not only prevent ribosomal proteins from engaging in illicit interactions and aggregation but also promote their nuclear import and/or assembly into pre-ribosomal particles. Tsr2 governs the nuclear transfer of Rps26 (eS26) from its importin to the 90S pre-ribosomal particle^14^. On the other hand, the ankyrin-repeat protein Yar1 protects Rps3 (uS3) from aggregation and may accompany Rps3 into the nucleus^9^,^34^, while the transport adaptor Syo1 mediates the synchronized co-import of Rpl5 (uL18) and Rpl11 (uL5; refs 8, 11). Intriguingly, both Syo1 and Yar1 recognize the N-terminal regions of Rpl5 (amino acids 2–20) and Rps3 (amino acids 14–29; refs 11, 34). In addition, the predicted WD-repeat β-propeller proteins Rrb1 and Sqt1 are proposed chaperones of Rpl3 (uL3) and Rpl10, respectively^35^,^36^,^37^,^38^. Rrb1, which is a mostly nucleolar protein, binds to Rpl3 and its overexpression leads to nuclear accumulation of Rpl3 (refs 36, 37). Moreover, Rrb1 redistributes from the nucleolus to the cytoplasm upon inhibition of translation, altogether suggesting that Rrb1 may already bind to Rpl3 in the cytoplasm^36^. Rpl3 associates very early with pre-60S subunits and is composed of a globular domain, which is positioned on the solvent-side surface of the LSU in close proximity of the sarcin-ricin loop, from which the N-terminal extension (amino acids 2–36) and the internal loop (W-finger; amino acids 221–273) emanate deep into the central core of the LSU^1^,^2^ (Supplementary Fig. 1). Increased dosage of Sqt1 was shown to suppress the growth defect conferred by overexpression of the N-terminal 64 amino acids of Rpl10 (ref. 35), thus, together with further genetic and biochemical data^35^,^38^, indicating that Sqt1 may recognize the N-terminal extension preceding Rpl10's conserved globular domain. Rpl10 is sandwiched between helices H38 (A-site finger) and H89 (refs 1, 2), therefore being located on the opposite side of the aminoacyl-tRNA accommodation corridor than Rpl3 (Supplementary Fig. 1). In addition, the internal loop (P-site loop; amino acids 102–112) of Rpl10, which contacts the P-site tRNA, controls, in cooperation with the N-terminal ‘hook' residues that reversibly interlock into H89, the rotational states of the ribosome during the elongation cycle^39^,^40^. Moreover, the P-site loop is required for the release of the anti-association factor Tif6 from pre-60S subunits^2^,^41^,^42^. The release of Tif6 is a pre-requisite for Lsg1-mediated recycling of Nmd3 (refs 30, 38, 43), which constitutes the final step of the cytoplasmic pre-60S maturation cascade^30^.

Given that Syo1 and Yar1 recognize the N-terminal regions of Rpl5 and Rps3 (refs 11, 34), we set out to explore the intuitive possibility that dedicated chaperones capture ribosomal proteins at the earliest possible moment in a co-translational manner. Here we also show that both Rrb1 and Sqt1 interact with the very N-terminal residues of Rpl3 and Rpl10, respectively. In line with our hypothesis, all four of these dedicated chaperones have the capacity to recognize their nascent ribosomal protein clients co-translationally. Moreover, the binding mode of the N-terminal residues of Rpl10 (L10-N) by the eight-bladed WD-repeat β-propeller of Sqt1, as revealed by X-ray crystallography, allows establishing a refined model for the final pre-60S maturation events that lead to the stable incorporation of Rpl10 and the release of Nmd3.

## Results

### Rrb1 and Sqt1 recognize the N termini of Rpl3 and Rpl10

**Figure 1:**
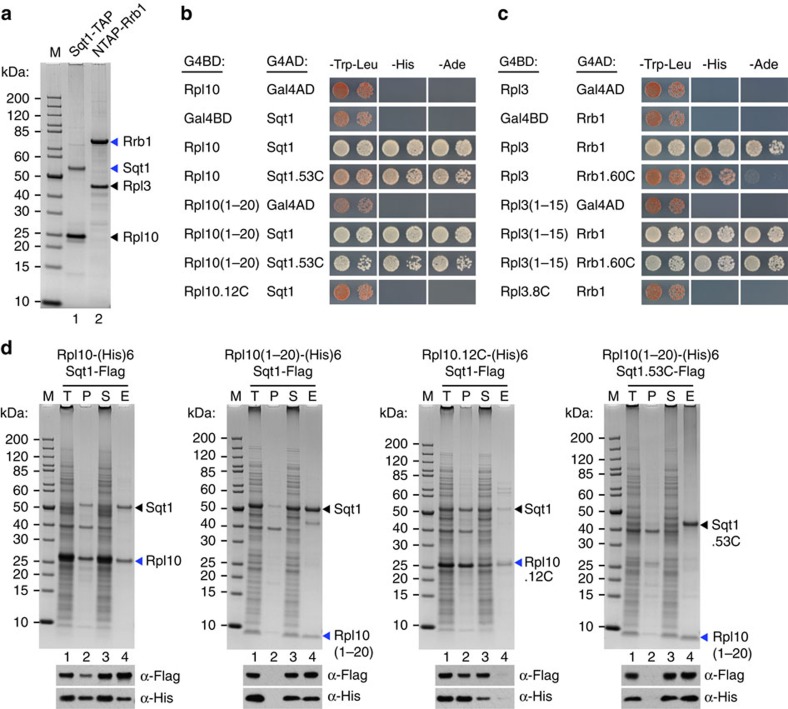
Sqt1 and Rrb1 recognize the N-terminal residues of Rpl10 and Rpl3, respectively. (**a**) Sqt1 and Rrb1 are exclusively associated with Rpl10 and Rpl3. TAP of C-terminally TAP-tagged Sqt1 (Sqt1-TAP, lane 1) and N-terminally TAP-tagged Rrb1 (NTAP-Rrb1, lane 2) from yeast cell lysates. Final EGTA eluates were analysed by SDS–polyacrylamide gel electrophoresis (PAGE) and Coomassie staining. (**b**) Y2H interaction between Rpl10 and Sqt1. Note that the Sqt1.53C protein lacks amino acids 1–52 and thus essentially contains the WD-repeat β-propeller domain of Sqt1. Rpl10.12C corresponds to an Rpl10 variant starting with amino acid 12. (**c**) Y2H interaction between Rpl3 and Rrb1. Note that the Rrb1.60C protein lacks amino acids 2–59 and thus contains the WD-repeat β-propeller domain, including a predicted N-terminal α-helix, of Rrb1 (Supplementary Fig. 2d). Rpl3.8C corresponds to an Rpl3 variant starting with amino acid eight. (**d**) *In vitro* binding assay between Rpl10 and Sqt1. The indicated C-terminally (His)_6_-tagged Rpl10 and C-terminally Flag-tagged Sqt1 variants were co-expressed in *E. coli* and purified via Ni-affinity purification. Proteins were revealed by SDS–PAGE and Coomassie staining (top) or by western blot analysis using anti-Flag (Sqt1-Flag variants) and anti-His (Rpl10-(His)_6_ variants) antibodies (bottom). T, total extract (lane 1); P, pellet fraction (insoluble proteins, lane 2); S, soluble extract (lane 3); E, imidazole eluate (lane 4); M, molecular weight standard. The bands highlighted by blue arrowheads correspond to the different Rpl10 variants used as baits for the purifications. Black arrowheads indicate the position of Sqt1-Flag and Sqt1.53C-Flag. Note that the third panel can be considered as a reference for the background binding of Sqt1-Flag to the Ni-NTA agarose resin.

To address whether Rrb1 and Sqt1 are exclusively associated with Rpl3 and Rpl10, respectively, we performed tandem-affinity purification (TAP) of NTAP-Rrb1 (NTAP, proteinA-TEV-CBP-Flag), expressed from a monocopy plasmid under the control of its cognate promoter in an *rrb1* null strain, and genomically expressed Sqt1-TAP. Importantly, both *SQT1*-TAP and NTAP-*RRB1*, unlike the genomic *RRB1*-TAP strain (see also ref. 37), were completely functional as judged by their capacity to confer wild-type growth (Supplementary Fig. 2a,b). Notably, we observed that mild overexpression of Rpl3 weakly suppressed the slow-growth phenotype of *rrb1*-TAP mutant cells (Supplementary Fig. 2c). In agreement with previous Rrb1 purifications (Rrb1-HA and Rrb1-TAP)^36^,^37^, NTAP-Rrb1 showed robust co-purification of Rpl3 (Fig. 1a). In affirmation of the proposed role of Sqt1 as a specific chaperone of Rpl10, we obtained good co-enrichment of Rpl10 when purifying the Sqt1-TAP bait (Fig. 1a). Unlike the dedicated transport adaptor Syo1, which binds simultaneously to the ribosomal proteins Rpl5 and Rpl11 (ref. 11), we did not observe any association of additional ribosomal proteins, besides Rpl3 or Rpl10, or biogenesis factors with purified Rrb1 or Sqt1.

To determine the binding site on Rpl3 and Rpl10 that is recognized by Rrb1 and Sqt1, respectively, we used yeast two-hybrid (Y2H) interaction assays. This analysis revealed that Sqt1, as already indicated by previous genetic and biochemical experiments^35^,^38^, binds to the first 64 amino acids of Rpl10 (data not shown). Progressive C-terminal shortening showed that amino acids 1–20 of Rpl10 are sufficient to mediate the interaction with Sqt1 (Fig. 1b). Likewise, amino acids 1–15 of Rpl3 are sufficient to yield a robust Y2H interaction with full-length Rrb1 (Fig. 1c). Deletion of the N-terminal residues from Rpl10 (12C construct, deletion of amino acids 3–11) and Rpl3 (8C construct, deletion of amino acids 2–7) abolished the interaction with Sqt1 and Rrb1, respectively (Fig. 1b,c), revealing that the N-terminal region is in both cases strictly required for the interaction. Further Y2H assays showed that the predicted WD-repeat β-propeller domains of Sqt1 (53C construct, deletion of amino acids 1–52) and Rrb1 (60C construct, deletion of amino acids 2–59) (Supplementary Fig. 2d), which support wild-type growth (Supplementary Fig. 2e,f), are sufficient to mediate the interaction both with full-length or the N-terminal residues of Rpl10 and Rpl3, respectively (Fig. 1b,c). To corroborate the Y2H data, we turned to *in vitro* binding assays. Since full-length Rpl10 was expressed as an insoluble protein in *Escherichia coli* (data not shown), we co-expressed C-terminally (His)_6_-tagged Rpl10 with Sqt1-Flag. Subsequent Ni-affinity purification of Rpl10-(His)_6_ resulted in an efficient co-purification of Sqt1-Flag (Fig. 1d). Further binding assays confirmed that the N-terminal residues of Rpl10 (L10-N) are required and sufficient for a robust interaction both with full-length Sqt1 and its β-propeller domain. Owing to inefficient expression of soluble Rrb1 in *E. coli*, it was, however, not possible to investigate the Rrb1-Rpl3 interaction by *in vitro* binding assays. In agreement with the N-terminal residues of Rpl10 and Rpl3 being the major binding determinants, overexpression of Sqt1 or Rrb1 efficiently suppressed the growth defect associated with the expression of L10-N (amino acids 1–20) and L3-N (amino acids 1–23) yEGFP fusion proteins, respectively (Supplementary Fig. 3a). In support of this genetic finding, these fusion proteins could be specifically co-purified *in vivo* with the Sqt1-TAP or the NTAP-Rrb1 bait, respectively (Supplementary Fig. 3b). Finally, *in vivo* co-purification of Rpl10-2xHA, Rpl3-2xHA and their N-terminal deletion variants with Sqt1-TAP and NTAP-Rrb1, respectively, revealed that the N-terminal residues were strictly required for the interaction (Supplementary Fig. 3c). We conclude that the β-propeller domains of Sqt1 and Rrb1, which harbour the essential function of these proteins, recognize the very N-terminal residues of Rpl10 and Rpl3. Notably, these N-terminal residues reside in both cases in the interior of the 60S ribosome and form extensive contacts with rRNA (Supplementary Fig. 1).

### The top surface of the Sqt1 β-propeller accommodates Rpl10

Next, we wished to assess the structural basis of the Sqt1–Rpl10 interaction in order to better illuminate the role of Sqt1 during incorporation of Rpl10 into pre-60S subunits. To this end, we also performed *in vitro* binding assays with the orthologous proteins from the thermophilic, filamentous ascomycete *Chaetomium thermophilum* (*ct*), whose proteins often exhibit improved biochemical properties^44^. Both *ct*Sqt1 and *ct*Sqt1.52C (deletion of amino acids 2–51), which fully complement the absence of Sqt1 *in vivo* (Supplementary Fig. 2e), could be efficiently co-purified with *ct*Rpl10 or its N-terminal 20 residues (Supplementary Fig. 4).

**Figure 2:**
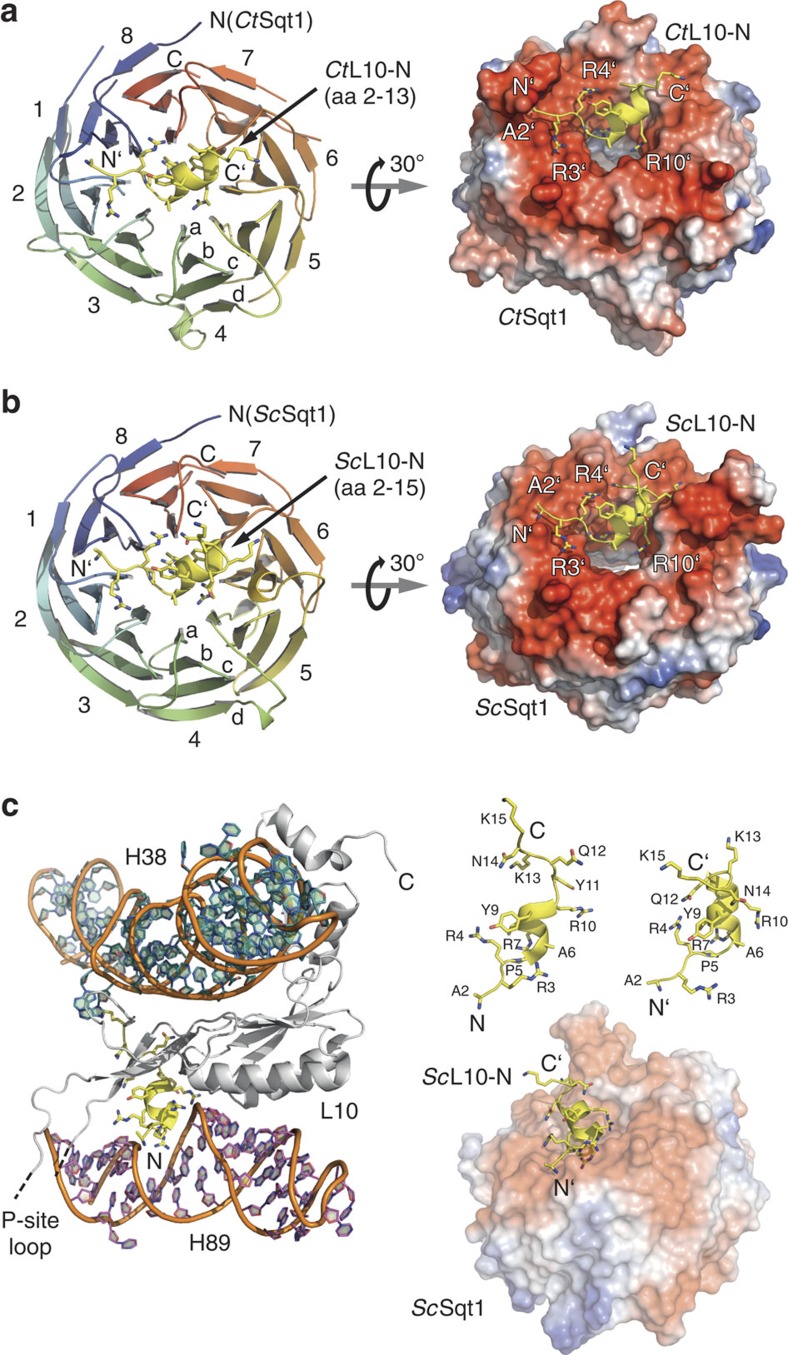
Crystal structures of the eight-bladed WD-repeat β-propeller domain of Sqt1 with bound L10-N from *S. cerevisiae* and *C. thermophilum*. (**a**) Crystal structure of *ct*Sqt1.52C (residues 52–533) with bound *ct*L10-N (residues 2–13). Cartoon representation showing *ct*Sqt1.52C in rainbow colours from N- to C terminus and *ct*L10-N in yellow with side chains (left panel). The eight-bladed WD-repeat β-propeller is shown in its top view. Assignment of the top and bottom surface as well as numbering of the propeller blades (1–8) and labelling of the β-strands within each blade (a–d) is according to the conventional definition for WD-repeat β-propellers. N- and C termini are indicated. Electrostatic properties of the top surface of *ct*Sqt1.52C with bound L10-N in yellow (right panel). (**b**) Crystal structure of *Sc*Sqt1.53C (residues 53–431) with bound *Sc*L10-N (residues 2–15). Cartoon representation (left panel) and electrostatic properties (right panel) of *Sc*Sqt1.53C with bound *Sc*L10-N in yellow. Labels and colouring is as in **a**. (**c**) Comparison of the interaction modes of *Sc*L10-N with helix H89 of the 25S rRNA and with *Sc*Sqt1, respectively. Cartoon representation of Rpl10 bound to H38 and H89 of the 25S rRNA as observed in the mature 60S subunit (PDB 3U5I and 3U5H for Rpl10 and 25S rRNA, respectively)^1^ (left panel) and bound to *Sc*Sqt1.53C (right panel). The N-terminal residues of Rpl10 (amino acids 2–15) are shown in yellow with side chains, the remainder of Rpl10 in grey, and bases of H38 in turquoise and of H89 in purple (phosphate backbones of H38 and H89 are shown in orange). Sqt1 is shown in its surface representation with electrostatic properties. The upper right part shows a comparison of the L10-N peptide in the ribosome-bound (left) and Sqt1-bound (right) state.

As a first step towards the elucidation of the recognition mode of the N-terminal residues of Rpl10 by the predicted β-propeller domain of Sqt1 at the atomic level, we independently determined the crystal structure of *ct*Sqt1 at 1.94 Å by molecular replacement and of *ct*Sqt1.52C, lacking the dispensable N-terminal extension, at 1.5 Å resolution by single-anomalous dispersion (SAD) followed by molecular replacement using the *ct*Sqt1.52C Se-Met structure as the search model (Table 1). While the N-terminal extension could not be resolved, the crystal structures revealed that residues 52–533 of *ct*Sqt1 form a typical eight-bladed WD-repeat β-propeller (Supplementary Fig. 5a); thus, being composed of eight blades that each contain four β-strands and showing the characteristic ‘velcro' closure owing to the presence of the N-terminal β-strand as the outermost β-strand of the eighth blade. Subsequently, we could solve the structure of the *S. cerevisiae* Sqt1 WD-repeat β-propeller domain at 2.0 Å resolution by molecular replacement using the native *ct*Sqt1.52C structure as the search model (Supplementary Fig. 5a). Sqt1 and *ct*Sqt1 share a high degree of overall structural conservation and mainly differ in three *ct*Sqt1-specific insertions located in the loops connecting β-strands 1c-1d, 5c-5d and 7c-7d (Supplementary Figs 5b and 11). Analysis of the electrostatic properties revealed that both β-propeller structures notably contain a negatively charged top surface, whereas the bottom sides exhibit a charge-mixed surface (Fig. 2 and Supplementary Fig. 5a).

Next, we co-crystallized the *S. cerevisiae* and *C. thermophilum* complexes between the β-propeller domain of Sqt1 and L10-N, which were co-expressed in *E. coli* and co-purified via Rpl10(1–20)-(His)_6_ by Ni-affinity chromatography followed by size-exclusion chromatography. Co-structures could be determined at 1.6 Å (*S. cerevisiae*) and 1.7 Å (*C. thermophilum*) resolution by molecular replacement using the respective native Sqt1 structures as search models (Table 2). The additional electron density contained well-defined side chains and main-chain carbonyls that could be unambiguously assigned to residues 2–15 (*Sc*) and 2–13 (*Ct*) of L10-N (Fig. 2a,b and Supplementary Fig. 6). In both cases, the L10-N residues are accommodated in the negatively charged top surface of the β-propeller and appear as elongated peptide chains with Ala6 to Gln11/Cys12 forming an α-helical segment (Fig. 2a,b). The Sqt1/L10-N-binding interface comprises ∼700 Å^2^ of surface area and is established by intricate hydrogen bonding networks, salt bridges and two hydrophobic patches (Supplementary Fig. 7a,b). In the context of the 60S r-subunit^1^, the L10-N residues, which form a continuous peptide with an α-helical segment of two turns, interlock into helix H89 (Fig. 2c). Therefore, the configuration of L10-N at the ribosome is highly similar to that observed when accommodated by Sqt1 (Fig. 2c). Notably, Sqt1 shields all L10-N residues that will be later on involved in the interaction with H89 of the 25S rRNA.

**Figure 3:**
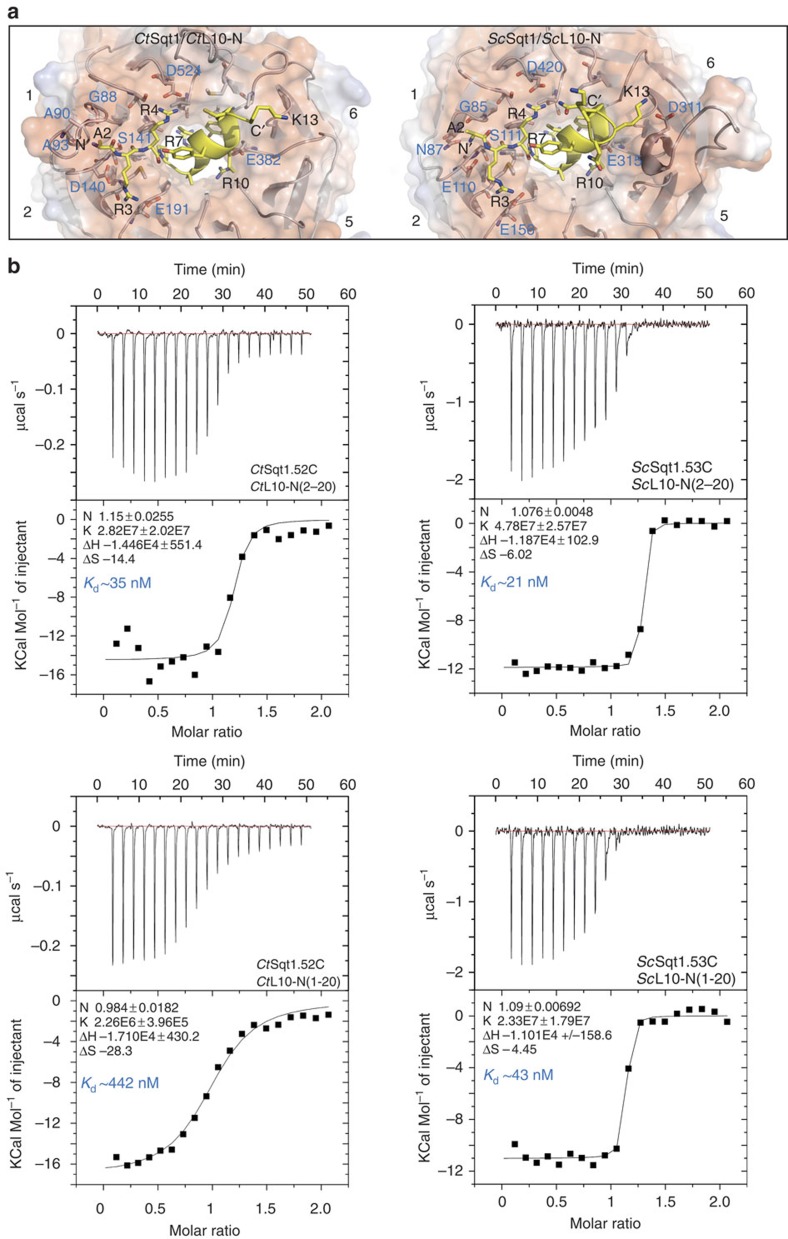
A thermophilic adaptation might sense the processing status of Rpl10's N-terminal methionine. (**a**) Close-up of the interaction between the L10-N residues and the WD-repeat β-propeller domain of *Ct*Sqt1 (left panel) and of *Sc*Sqt1 (right panel). Sqt1 is shown in its cartoon representation with superimposed electrostatic surface properties. The Sqt1 residues involved in the interaction are shown as sticks. L10-N residues (yellow) are shown in a mixed cartoon/stick representation. The relevant Sqt1 and L10-N residues are labelled in blue and black, respectively (for example, A2 for Ala2). N- and C termini of L10-N are indicated (N′ and C′). (**b**) Analysis of the L10-N interaction with the WD-repeat β-propeller domain of *Ct*Sqt1 and *Sc*Sqt1 by ITC. Shown are ITC measurements of *Ct*Sqt1.52C/*Sc*Sqt1.53C with *Ct*L10-N/*Sc*L10-N peptides either lacking (amino acids 2–20) or including Met1 (amino acids 1–20), as indicated in each panel. The upper part of each panel shows the raw injection heats (μcal s^−1^). The lower part of each panel displays the corresponding specific binding isotherms (Kcal mol^−1^ of injectant) plotted against the molar ratio. The measured interaction parameters are listed within the profiles and the approximate *K*_d_ is shown in blue.

Interestingly, there are subtle differences between the binding surfaces formed by *S. cerevisiae* and *C. thermophilum* Sqt1, which mainly affect the recognition of the N-terminal residue Ala2 and the C-terminal part of the L10-N peptide. In the case of *ct*Sqt1, the amino group of Ala2 is triangulated by hydrogen bonds involving the backbone carbonyls of Gly88, Ala90 and Ala93 (Fig. 3a). These residues are part of a thermophile-specific insertion within the surface loop connecting β-strands 1b and 1c, notably forming a narrow, cap-like binding pocket, which would not provide enough space for the accommodation of the N-terminal methionine (Met1). In the case of *S. cerevisiae* Sqt1, the amino group of Ala2 is held in place via interactions with the main-chain carbonyl of Gly85 and the side chains of Asn87, Glu110 and Ser111 (Fig. 3a). At the C-terminal end of the L10-N peptide, only Lys13 of *S. cerevisiae* engages in a contact, involving Asp311, with Sqt1. Since Met1 of Rpl10 is not present in the *S. cerevisiae* 60S structure^1^, we next addressed whether co-translational removal of the N-terminal methionine by the ribosome-associated methionine amino peptidase^45^,^46^ is a pre-requisite for the recognition of L10-N by Sqt1. To this end, we quantified the binding of Sqt1 to L10-N peptides, either containing (amino acids 1–20) or lacking Met1 (amino acids 2–20), by isothermal titration calorimetry (ITC; Fig. 3b). Sqt1 from *S. cerevisiae* showed only a slight preference for the L10-N peptide lacking Met1, as indicated by the dissociation constants (*K*_d_) of ∼21 and 43 nM, respectively. In the case of *ct*Sqt1, however, the presence of Met1 reduced the affinity for the L10-N peptide by about tenfold (*K*_d_ of ∼35 and ∼442 nM), but did not abolish the interaction completely. We conclude that Sqt1 forms a remarkably stable interaction with the N-terminal residues of Rpl10, which is, at least as observed for *ct*Sqt1, very sensitive to the presence of the N-terminal methionine.

### Negatively charged surface residues mediate Rpl10 binding

**Figure 4:**
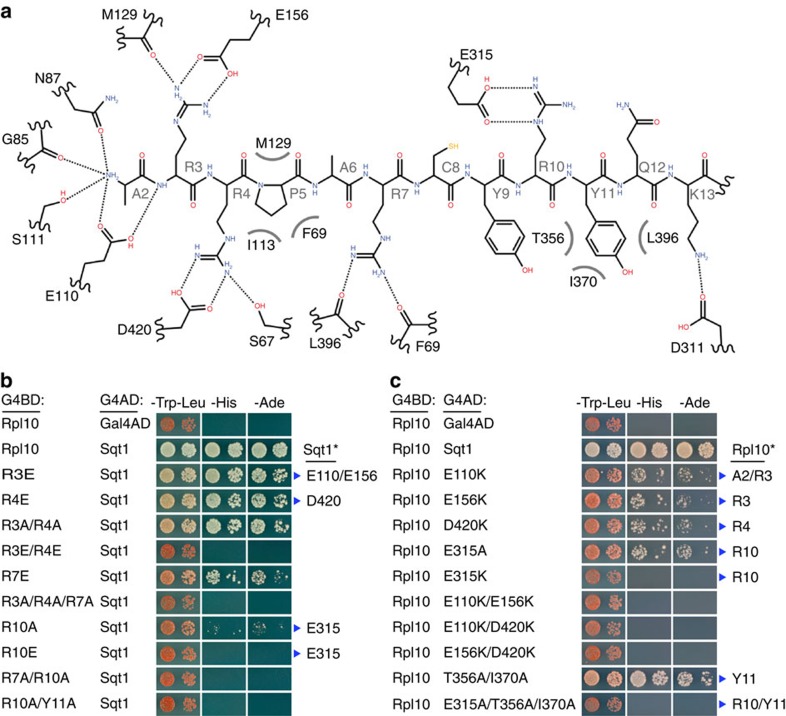
Ionic interactions are critical determinants of L10-N binding by Sqt1. (**a**) Representation of the mode of L10-N recognition by *Sc*Sqt1. The backbone and side chains of *Sc*L10-N (residues 2–13) are shown as an elongated peptide. L10-N residues are labelled in grey (e.g.,: A2 for Ala2). The Sqt1 residues that form interactions, either via their side chains or main-chain carbonyls, with the L10-N peptide are indicated. Dotted lines indicate ionic interactions or hydrogen bonds and grey, curved lines hydrophobic interactions. The interaction representation was created with Accelrys Draw 4.1. (**b**) Y2H interaction between Sqt1 and Rpl10 variants harbouring mutations within the N-terminal residues. The residues mutated in Rpl10 (for example, R3E for Arg3 to glutamate), as well as the Sqt1 residues they are contacting (blue arrowheads, Sqt1*), are indicated. (**c**) Y2H interaction between Rpl10 and mutant Sqt1 variants. The residues mutated in Sqt1 (for example, E110K for Glu110 to lysine), as well as the L10-N residues they are contacting (blue arrowheads, Rpl10*), are indicated. Single-letter abbreviations for the amino acid residues are as follows: A, Ala; D, Asp; E, Glu; I, Ile; K, Lys; R, Arg; T, Thr; and Y, Tyr.

Since the interaction between Sqt1 and the L10-N peptide involves many salt bridges and hydrogen bonds (Fig. 4a), we mainly focused on the arginine residues within the L10-N peptide (Arg3, 4, 7 and 10) and the four conserved negatively charged residues of Sqt1 (Glu110, Glu156, Glu315 and Asp420) in order to determine their contribution to the interaction by Y2H analyses. While mutation of Arg10 to glutamate or alanine abolished or already reduced the interaction with Rpl10, respectively, only combinations of simultaneous substitutions of Arg3, Arg4 and Arg7 abrogated or interfered with Sqt1 binding (Fig. 4b and Supplementary Fig. 7c). In agreement with this result, mutation of Sqt1 residue Glu315, which contacts Arg10, to lysine abolished the interaction between Sqt1 and Rpl10 (Fig. 4c). Moreover, similar reductions in Y2H interaction were observed for the E110K, E156K, D420K and E315A Sqt1 variants; and, as above, only combinations of Glu110, Glu156 and Asp420 substitutions eliminated or reduced the interaction with Rpl10 (Fig. 4c and Supplementary Fig. 7d). We conclude that the Arg10–Glu315 interaction is the main binding determinant and that the Arg3–Glu110/Glu156 and Arg4–Asp420 interactions are individually not strictly required for but clearly contribute to binding.

**Figure 5:**
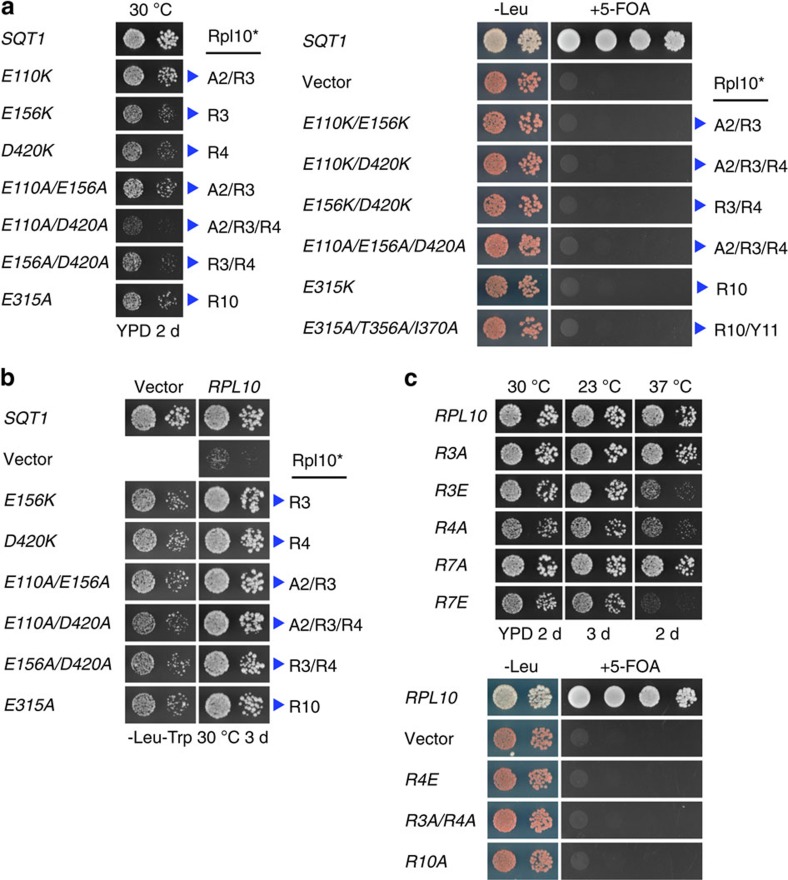
Overexpression of Rpl10 bypasses the requirement for the essential Sqt1. (**a**) *In vivo* phenotypes of cells expressing Sqt1 variants that affect the interaction with Rpl10. Growth phenotypes of viable *sqt1* mutants on YPD plates that were incubated for 2 days at 30 °C (left panel). The lethality of *sqt1* alleles that abolish the interaction with Rpl10 was scored on plates containing 5-fluoroorotic Acid (5-FOA), which were incubated for 3 days at 30 °C (right panel). The residues mutated in Sqt1, as well as the L10-N residues they are contacting (blue arrowheads, Rpl10*), are indicated. (**b**) Overexpression of Rpl10 suppresses the slow-growth phenotype of cells expressing Sqt1 variants that affect the interaction with Rpl10 and even rescues the absence of Sqt1. Cells harbouring the *SQT1* wild-type allele or the indicated *sqt1* alleles were grown in the absence (vector) or presence of a multicopy plasmid expressing Rpl10 (*RPL10*) on SC-Leu-Trp plates that were incubated for 3 days at 30 °C. (**c**) *In vivo* phenotypes of cells expressing Rpl10 variants harbouring mutations within the N-terminal residues. The growth phenotypes of the indicated *rpl10* alleles were scored on YPD plates (viable mutants; upper panel), which were incubated as indicated, and on 5-FOA-containing plates (lethal mutants; lower panel), which were incubated for 3 days at 30 °C.

**Figure 6:**
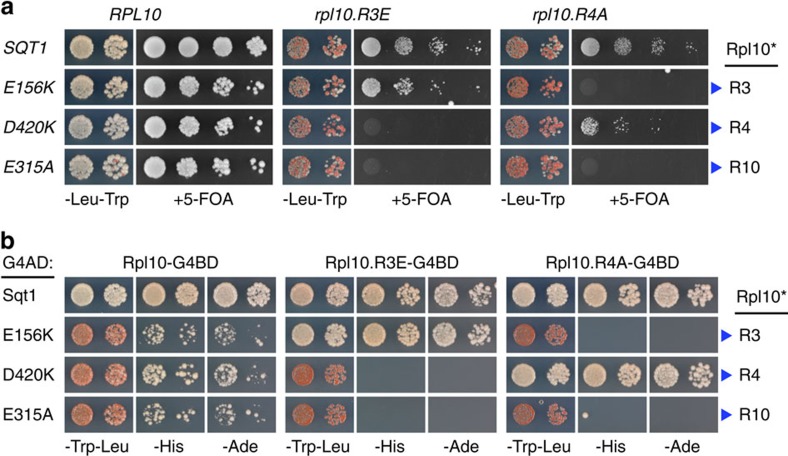
The essential function of Sqt1 consists in Rpl10 binding. (**a**) Allele-specific synthetic lethality between interaction surface mutants of *sqt1* and *rpl10*. The growth phenotypes of cells harbouring the *SQT1* wild-type allele or the indicated *sqt1* alleles in combination with the *RPL10* wild-type allele or the *rpl10.R3E* and *rpl10.R4A* allele were scored on 5-FOA-containing plates, which were incubated for 4 days at 30 °C. The mutated Sqt1 residues, as well as the L10-N residues they are contacting (blue arrowheads, Rpl10*), are indicated. (**b**) Allele-specific abrogation of the interaction between the above interaction surface mutants of *sqt1* and *rpl10*. Y2H interactions were assessed for combinations between Sqt1 or the indicated Sqt1 variants and Rpl10 or the Rpl10.R3E and Rpl10.R4A mutant variant.

In order to assess the functional relevance of the Y2H interaction data, we next determined the *in vivo* phenotypes of the *sqt1* mutations that affect interaction with Rpl10 by growth assays. In agreement with the above binding studies, the Glu315 to lysine substitution was the only *sqt1* single mutation that did not support growth, while combinations of alanine or lysine substitutions of Glu110, Glu156 and Asp420 were required to reduce or abolish growth (Fig. 5a and Supplementary Fig. 8a). In support of Rpl10 binding being the exclusive cellular role of Sqt1, overexpression of Rpl10 from a multicopy plasmid fully suppressed the slow-growth phenotypes of *sqt1* mutants (Fig. 5b), while we observed partial growth restoration in case of the lethal *sqt1* alleles (Supplementary Fig. 8b). Strikingly, Rpl10 overexpression even conferred very weak growth to cells lacking Sqt1 (Fig. 5b and Supplementary Fig. 8b). In agreement with a chaperone function of Sqt1, we observed that the solubility of newly synthesized Rpl10-2xHA, expressed for 20 min from a copper-inducible promoter, is strongly reduced in *sqt1.E315A* and *sqt1.E110A/D420A* mutant cells (Supplementary Fig. 9). Since the N-terminal residues of Rpl10 make extensive contacts with rRNA and have been implicated in coordination of tRNA movement (Supplementary Fig. 1; ref. 39), the effects of their mutation on growth cannot simply be correlated to their contribution to Sqt1 binding. Accordingly, the substitution of Arg4 to glutamate and the double substitution of Arg3/Arg4 to alanine, which only slightly reduced the interaction with Sqt1 (Fig. 4b), resulted in a lethal phenotype (Fig. 5c). Nevertheless, it was possible to obtain slow-growing *rpl10* mutants, for example, *rpl10.R3E* and *rpl10.R4A* (Fig. 5c and Supplementary Fig. 8c), which were suitable to be exploited for the determination of synthetic lethal interactions with *sqt1* alleles. In validation of the co-crystal structure and the above Y2H data, we only observed synthetic lethal phenotypes when the combined *sqt1* and *rpl10* mutations affected different interaction pairs (Fig. 6a). While the *sqt1.E156K* mutation, which interferes with Arg3 interaction, was selectively synthetically lethal with the Arg4 to alanine substitution within L10-N, there was no synthetic growth defect when this *sqt1* allele was combined with the Arg3 to glutamate substitution. Likewise, only the combination of the *sqt1.D420K* allele, which abrogates Arg4 binding, with the Arg3 to glutamate, but not the Arg4 to alanine, substitution resulted in lethality. Finally, the *sqt1.E315A* allele, which abolishes interaction with Arg10, was synthetically lethal with both *rpl10* mutations. These allele-specific effects were even more striking at the level of the Y2H interaction (Fig. 6b). As expected, the R3E/D420K, R3E/E315A, R4A/E156K and R4A/E315A combinations abolished the Rpl10–Sqt1 interaction. However, reversion or elimination of the charge repulsion in the case of the R3E/E156K and R4A/D420K pairs resulted in a substantially improved interaction compared with the Y2H binding of wild-type Rpl10 to the E156K and D420K variants.

### Chaperones are recruited to nascent ribosomal proteins

**Figure 7:**
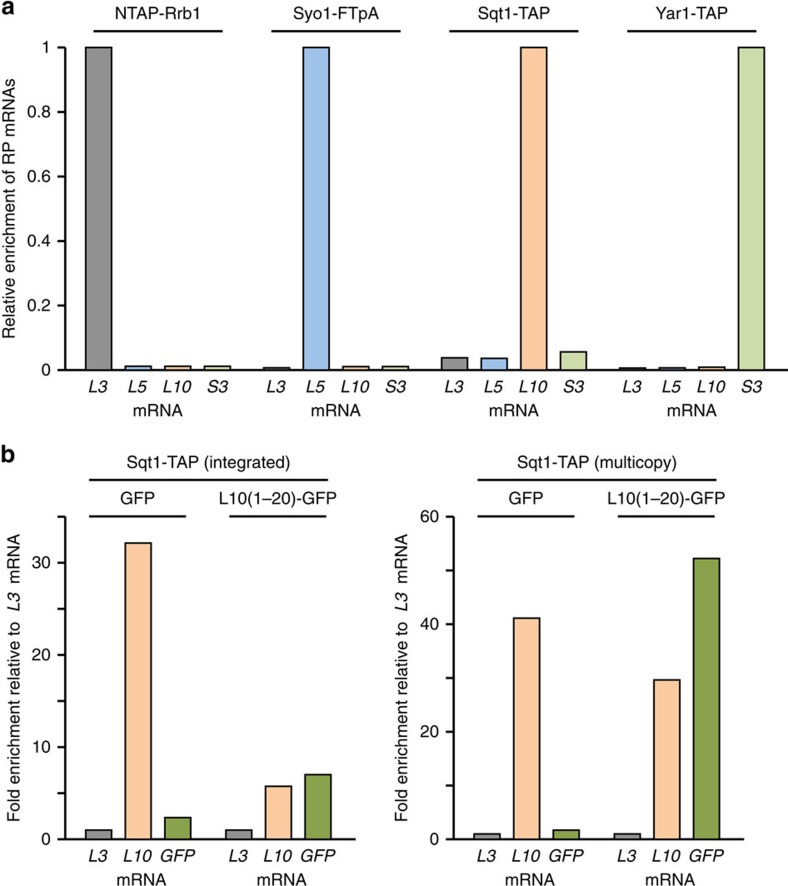
Chaperones are recruited to nascent ribosomal proteins. (**a**) The chaperone proteins were affinity purified (IgG-Sepharose pull-down) from extracts of cycloheximide-treated cells and the associated RNA was isolated from the TEV eluates. Each of the four chaperone purifications (NTAP-Rrb1, Syo1-FTpA, Sqt1-TAP, and Yar1-TAP) was assessed for their content of the four ribosomal protein (RP) mRNAs (*RPL3*, *RPL5*, *RPL10* and *RPS3*) by real-time qRT–PCR. The data from one representative experiment are expressed as the relative enrichment of the specifically co-purified RP mRNA in each of the four chaperone purifications (see Methods section for details). For each cDNA, real-time qPCRs were performed in triplicates. A highly reproducible data set was obtained in an independent series of chaperone purifications. (**b**) The N-terminal residues of Rpl10 are sufficient to target Sqt1 to the nascent Rpl10(1–20)-yEGFP fusion protein. Sqt1-TAP, either expressed from the genomic locus (left panel) or from a multicopy plasmid (right panel) was affinity purified (IgG-Sepharose pull-down) from extracts of cells where expression of either the yEGFP (GFP) control protein or the Rpl10(1–20)-yEGFP [L10(1–20)-GFP] fusion protein has been induced from the *CUP1* promoter for 10 min with 500 μM copper sulfate. The Sqt1-TAP purifications were assessed for their content of the *RPL3*, *RPL10* and yEGFP (GFP) mRNAs by real-time qRT–PCR. The data from one representative experiment are expressed as the fold enrichment relative to the *RPL3* mRNA. For each cDNA, real-time qPCRs were performed in triplicates. Note that the bar graphs of the left and right panel of this figure are at a different scale.

Given that each of the distinct chaperones interacts with the N-terminal residues of the respective ribosomal protein client (Rpl3, amino acids 1–15; Rpl5, amino acids 2–20; Rpl10, amino acids 2–13; and Rps3, amino acids 14–29; Figs 1 and 2; refs 11, 34), we sought to explore the intuitive possibility that the chaperones are already recruited to nascent ribosomal proteins as these are translated from their mRNA. To this end, we purified each of the four different chaperones by immunoglobulin G (IgG)-sepharose pull-down from cell extracts of yeast cells that were, before harvesting, treated with cycloheximide, which blocks translation elongation, and thus preserves the translating ribosomes on the mRNAs (see Methods section). The purified chaperone and any associated molecules were then released from the IgG-sepharose beads by tobacco etch virus (TEV) protease cleavage and the RNA was subsequently isolated. To unambiguously reveal the specific presence of the corresponding ribosomal protein encoding mRNA, each of the four chaperone purifications was assessed for their content of the four ribosomal protein mRNAs (*RPL3*, *RPL5*, *RPL10* and *RPS3*) by real-time quantitative reverse transcription PCR (real-time qRT–PCR). This analysis clearly showed that each of the four chaperones was specifically co-purifying the mRNA encoding its ribosomal protein client (Fig. 7a). While we observed a roughly 100-fold enrichment of the specific ribosomal protein mRNA in the case of Rrb1, Syo1 and Yar1, the enrichment of the *RPL10* mRNA in the Sqt1 purification was clearly evident, albeit less pronounced (∼25-fold). Moreover, we could also detect co-purification of the specific ribosomal protein encoding mRNAs when cycloheximide was omitted (Supplementary Fig. 10a); thus, ruling out that the observed co-purification was simply due to an association with the nascent ribosomal proteins on elongation-blocked ribosomes during the 5-min period of the cycloheximide treatment. Finally, we expressed L10-N (amino acids 1–20) and L3-N (amino acids 1–23) yEGFP fusion constructs for 10 min from a copper-inducible promoter, followed by cycloheximide treatment, and assessed the content of the yEGFP mRNA in the Sqt1-TAP and NTAP-Rrb1 purification, respectively. While the co-translational association with the specific ribosomal protein encoding mRNA was reduced, the L10-N-yEGFP and L3-N-yEGFP were clearly enriched compared with the yEGFP control mRNA (Fig. 7b and Supplementary Fig. 10b). Notably, the selective co-purification of the L10-N-yEGFP mRNA was more evident when Sqt1-TAP was overexpressed from a multicopy plasmid (Fig. 7b), suggesting that genomically expressed Sqt1-TAP was efficiently titrated by the newly synthesized L10-N-yEGFP fusion protein (see also Supplementary Fig. 3b). We conclude that each of the four chaperones has the capacity to recognize its specific ribosomal protein substrate in a co-translational manner. The high affinity of the interaction between Sqt1 and L10-N (*K*_d_ ∼20 nM) suggests that chaperone recruitment to nascent ribosomal proteins may represent the default setting of this process *in vivo*.

## Discussion

Recent evidence has revealed that the dedicated chaperones Syo1 and Yar1 interact with the N-terminal region of their ribosomal protein partners Rpl5 (amino acids 2–20) and Rps3 (amino acids 14–29; refs 9, 11, 34). In this study, we have shown that the proposed chaperones Rrb1 and Sqt1 recognize the very N-terminal residues of Rpl3 (amino acids 1–15) and Rpl10 (amino acids 2–13), respectively. In the case of Sqt1, we were able to decipher the mode of L10-N peptide binding by X-ray crystallography (Figs 2 and 3). These structural analyses showed that the C-terminal domain of Sqt1, which is preceded by a non-essential N-terminal extension of ∼50 amino acids, folds into an eight-bladed WD-repeat β-propeller. While WD-repeat β-propellers represent the most abundant domain type in the *S. cerevisiae* proteome, eight-bladed β-propellers are far less prevalent than the archetypal seven-bladed WD-repeat β-propellers^47^. Even though WD-repeat β-propellers share, at a first glance, a striking overall structural similarity, they serve as astonishingly versatile interaction platforms^47^. As revealed by co-structures, bound peptides are in most cases accommodated on the top surface of the β-propellers and the residues that generally mediate the interaction with the peptides are located at the beginning of the a (mostly two or more residues) and at the end of the b (mostly one residue) β-strands of the WD repeats (see Fig. 2a and its legend for the definition of the a and b β-strands)^47^. In this sense, the binding of the L10-N peptide by the top surface of Sqt1 follows the above-described predominant mode of interaction by involving, as also in part experimentally validated, one to two residues per WD repeat that lie at the beginning of the a β-strands (six out of eight WD repeats) and mostly one residue per WD repeat at the end of the b β-strands (four out of eight WD repeats; Supplementary Fig. 11).

Our data also provide additional evidence that Sqt1 can be considered as a chaperone of Rpl10. First, purification of Sqt1-TAP from yeast cells yielded a substantial and exclusive co-enrichment of non-ribosome-associated Rpl10. Second, genetic experiments showed that overexpression of Rpl10 can bypass the requirement for Sqt1, and that, as revealed by allele-specific synthetic lethal interactions, the essential function of Sqt1 resides in its capacity to interact with Rpl10. Third, Sqt1 is required for the soluble expression of Rpl10 in yeast cells. Fourth, all L10-N residues, notably including the four prominent arginines (Arg3, 4, 7 and 10), that are involved in the interaction with helix H89 of the 25S rRNA are covered by Sqt1, and are thus prevented from engaging in illicit interactions with cytoplasmic polyanions. Interestingly, such an RNA mimicry function of Sqt1 is reminiscent of how the adenylate kinase Fap7 blocks the rRNA-binding site of Rps14 (ref. 48). Taken together, we propose that Sqt1 may protect Rpl10 from aggregation before and/or promote its incorporation into almost mature pre-60S subunits in the cytoplasm.

**Figure 8:**
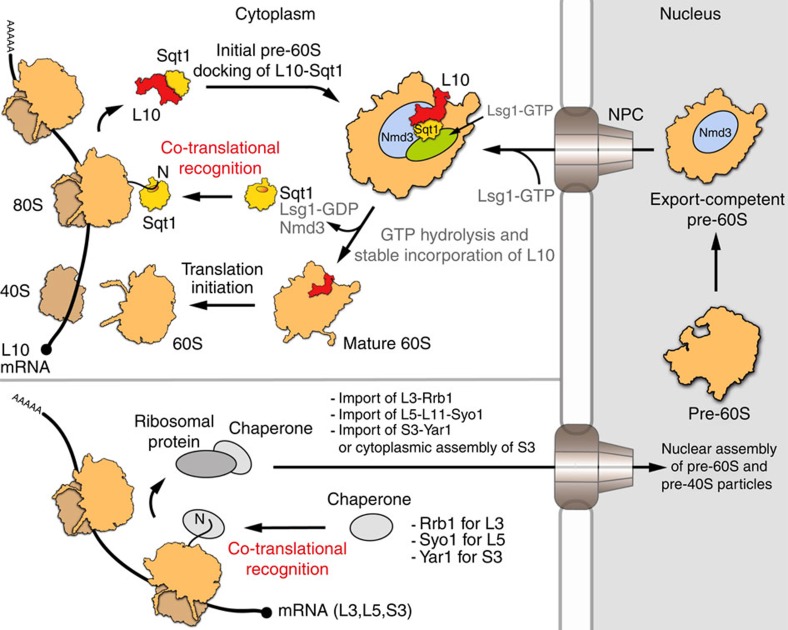
Model highlighting the co-translational capturing of selected ribosomal proteins by their dedicated chaperones. Simplified model for the stable incorporation of Rpl10 into cytoplasmic pre-60S subunits (upper left part). The chaperone Sqt1 recognizes the N-terminal residues of Rpl10 as these emerge from translating ribosomes and the Sqt1–Rpl10 complex is released into the cytoplasm upon translation termination. We propose that initial docking of Sqt1-bound Rpl10 onto Lsg1-defined pre-60S subunits involves Rpl10 surfaces that are not shielded by Sqt1 and likely occurs at pre-60S sites that are not masked by Nmd3 (see Discussion section). Subsequently, the activity of the GTPase Lsg1 entails structural rearrangements that promote the release of Nmd3 and the stable incorporation of Rpl10, thus leading to the generation of mature 60S subunits that can engage in translation initiation. General model for the co-translational capturing of ribosomal proteins by their specific chaperone partners (lower left part). This study has revealed that the chaperones Rrb1, Syo1 and Yar1 are also recruited to their distinct ribosomal protein clients (Rpl3, Rpl5 and Rps3), as these are synthesized from their mRNAs by the ribosome. While the Rrb1-Rpl3 and Syo1-Rpl5-Rpl11 complexes are imported into the nucleus where these ribosomal proteins assemble into pre-60S subunits, it is not yet clear whether Yar1 travels together with Rps3 into the nucleus or promotes pre-40S assembly of Rps3 in the cytoplasm. After extensive nuclear maturation (right part), pre-60S particles gain export competence upon recruitment of Nmd3, which is recognized by the exportin Crm1, and travel across the nuclear pore complex to the cytoplasm.

Elegant work from the Johnson laboratory has revealed that Sqt1, Rpl10 and the GTPase Lsg1 are required for the release of the export adaptor Nmd3 from cytoplasmic pre-60S subunits, and that, moreover, Sqt1 is only significantly associated with Nmd3- and Lsg1-containing pre-60S subunits upon overexpression of dominant-negative Lsg1(K349T) mutant protein^38^,^43^. Accordingly, it has been proposed that Lsg1 promotes and couples Nmd3 release to Rpl10 docking on a transient pre-60S intermediate containing Lsg1, Nmd3 and the Sqt1-bound Rpl10 (ref. 38). The structural insight provided by this study, in combination with the 60S crystal structure and the recent identification of the rRNA-binding sites of Nmd3 (refs 1, 49), allows proposing a refined model for the above-mentioned pre-60S maturation events. Since the main rRNA-binding sites of Nmd3 (H38, H69 and H89; ref. 49) and Rpl10 (H38 and H89; ref. 1) are partially overlapping, Nmd3 and Rpl10 cannot bind simultaneously with their maximal affinities to pre-60S subunits. Therefore, Rpl10, whose access to H89 is blocked due to Sqt1 being bound to its N-terminal residues, must initially be recruited via interaction sites that are not masked by Nmd3. Potential candidate sites that could mediate initial Rpl10 binding consist of the base of H38 and the last α-helix within the eukaryote-specific C-terminal extension of Rpl5 (ref. 1). While the base of H38 makes extensive contacts with different regions along Rpl10, the α-helix of Rpl5 interacts with the C-terminal α-helix of the eukaryote-specific extension of Rpl10 (amino acids 169–221; Supplementary Fig. 1). However, it remains to be determined whether or how the initial docking or stable incorporation of Rpl10 may contribute to the recently described rotation of the 5S RNP and H38 into their final position^50^, since the Lsg1-defined cytoplasmic pre-60S subunits may have already adopted this conformation. Upon initial binding of Rpl10, the GTPase Lsg1, either due to GTP binding or GTP hydrolysis, may then promote structural rearrangements that weaken the association of Nmd3, thereby allowing recognition of H89 by Rpl10, and thus facilitating the transfer of the N-terminal Rpl10 residues from Sqt1 into H89. Finally, these interconnected events would have entailed structural alterations that are only compatible with complete docking of Rpl10 and release of Nmd3 (for a simplified model, see Fig. 8).

Most notably, this study has revealed that dedicated chaperones have the capacity to recognize their distinct ribosomal protein partners as these are synthesized by the ribosome. Such an early recognition of ribosomal proteins represents an elegant mechanism to already predetermine the fate of the ribosomal proteins during their synthesis, thereby assuring their stable expression, correct sub-cellular targeting and, thus, correct assembly into pre-ribosomal particles (Fig. 8). However, this may not represent an obligatory step since overexpression of the respective ribosomal protein can bypass the requirement for its specific chaperone in the case of Sqt1, Syo1 and Yar1 (Fig. 5b; refs 9, 11). Given that importins can also fulfil in part these functions^32^, it is evident that not all ribosomal proteins will require specific chaperones or that they may even rely on alternative strategies, such as the fusion to an N-terminal ubiquitin moiety^51^. However, the association with a specific chaperone is an advantageous concept, as already evidenced by the Syo1-mediated coordination of nuclear co-import of Rpl5 and Rpl11 with 5S RNP assembly^11^,^52^. Taken together, a novel step of ribosome biogenesis, beginning as early as with the co-translational recruitment of specific chaperones to nascent ribosomal proteins, can be defined.

## Methods

### Yeast strains, yeast genetic methods and plasmids

The *S. cerevisiae* strains used in this study are listed in Supplementary Table 1; all strains, unless otherwise specified, are derivatives of W303. Deletion disruption and C-terminal tagging were performed according to standard procedures. Preparation of media, yeast transformation and genetic manipulations were according to established procedures. For the experiments involving induction of expression by addition of copper sulfate, media were prepared with copper-free yeast nitrogen base (FORMEDIUM). All recombinant DNA techniques were performed according to established procedures using *E. coli* DH5α for cloning and plasmid propagation. Codon-optimized (for *E. coli* expression) *C. thermophilum ct*SQT1 and *ct*RPL10 genes were generated by custom DNA synthesis (Eurofins). All cloned DNA fragments generated by PCR amplification were verified by sequencing. Plasmids used in this study are listed in Supplementary Tables 2 and 3.

### Y2H interaction analysis

For Y2H interaction assays, plasmids expressing bait proteins, fused to the Gal4 DNA-binding domain (G4BD), and prey proteins, fused to the Gal4 activation domain (G4AD), were co-transformed into reporter strain PJ69-4A. Y2H interactions were documented by spotting representative transformants in 10-fold serial dilution steps onto SC-Trp-Leu, SC-Trp-Leu-His (*HIS3* reporter) and SC-Trp-Leu-Ade (*ADE2* reporter) plates, which were incubated for 3 days at 30 °C. Growth on SC-Trp-Leu-His plates is indicative of a weak/moderate interaction, whereas only relatively strong interactions permit growth on SC-Trp-Leu-Ade plates.

### TAP and *in vitro* binding assays

Cells expressing Sqt1-TAP and NTAP-Rrb1 were grown at 30 °C in 4 l yeast extract peptone dextrose (YPD) medium to an optical density (OD_600_) of 2. Cell extracts were obtained by glass bead lysis with a Pulverisette (Fritsch). TAPs were performed in a buffer containing 50 mM Tris-HCl (pH 7.5), 100 mM NaCl, 1.5 mM MgCl_2_, 5% glycerol and 0.1% NP-40 as described^53^. The EGTA eluates were precipitated by the addition of TCA to a final concentration of 10% and, after an acetone wash, dissolved in 80 μl of 3 × SDS sample buffer. Protein samples were separated on NuPAGE 4–12% Bis-Tris 12-well gels (Novex), run in 1 × MES SDS running buffer, and subsequently stained with Brilliant Blue G Colloidal Coomassie (Sigma).

For *in vitro* binding assays between Rpl10-(His)_6_ and Sqt1-Flag or between *ct*Rpl10-(His)_6_ and *ct*Sqt1, proteins were co-expressed from pETDuet-1 (Novagen) in Rosetta(DE3) (Novagen) or BL21(DE3) (Novagen) *E. coli* cells, respectively. Cells were grown in 200 ml of lysogeny broth medium and protein expression was induced at an OD_600_ of ∼0.6–0.8 by the addition of isopropyl-β-D-thiogalactoside to a final concentration of 0.5 mM. After 5 h of growth at 30 °C, cells were harvested and stored at −80 °C. Cells were resuspended in 25 ml lysis buffer (50 mM Tris-HCl (pH 7.5), 200 mM NaCl, 1.5 mM MgCl_2_ and 5% glycerol) and lysed with a M-110L Microfluidizer (Microfluidics). The lysate (30 ml volume) was adjusted by the addition of 300 μl 10% NP-40 to 0.1% NP-40 (note that from here onwards all buffers contained 0.1% NP-40). An aliquot of 100 μl of total extract (sample T) was taken and mixed with 100 μl of 6 × loading buffer. The total extract was then centrifuged at 4 °C for 20 min at 14,000 r.p.m. The soluble extract was transferred to a 50-ml Falcon tube and, as above, an aliquot of 100 μl of soluble extract (sample S) was taken and mixed with 100 μl of 6 × loading buffer. The insoluble pellet fraction (sample P) was resuspended in 3 ml of lysis buffer, and 10 μl thereof were mixed with 90 μl of lysis buffer and 100 μl of 6 × loading buffer. The soluble extract (30 ml) was adjusted to 15 mM imidazole by adding 180 μl 2.5 M imidazole (pH 8). Upon addition of 250 μl of Ni-NTA Agarose slurry (Qiagen), samples were incubated for 2 h on a turning wheel at 4 °C and then applied to a 10-ml Poly-Prep column (Bio-Rad). The drained column was washed four times with 5 ml of lysis buffer containing 15 mM imidazole. Then, after addition of 1 ml lysis buffer containing 50 mM imidazole, the column was sealed and incubated for 2 min on a turning wheel at 4 °C. For elution, 1 ml of lysis buffer containing 250 mM imidazole was added, and the sealed column was again incubated for 2 min on a turning wheel at 4 °C. The eluate (sample E) was collected in a 1.5-ml Eppendorf tube, and 100 μl thereof were mixed with 100 μl of 6 × loading buffer. Protein samples (5 μl of samples T, P, S and E) were separated on NuPAGE 4–12% Bis-Tris 15-well gels (Novex), run in 1 × MES SDS running buffer and subsequently stained with Brilliant Blue G Colloidal Coomassie (Sigma). For western blot analysis, appropriate dilutions of the above samples were separated on Bolt 4–12% Bis-Tris Plus 15-well gels (Novex), run in 1 × MES SDS running buffer, and subsequently blotted onto nitrocellulose membranes (GE Healthcare).

To reveal the proteins of interest by western blot analysis, mouse monoclonal anti-FLAG (1:2,000–1:10,000; Sigma), anti-penta-His (1:500; Qiagen), anti-GFP (1:2,000; Roche), anti-HA (1:3,000; BAbCO) and anti-Rpl3 (1:2,000; J. Warner, Albert Einstein College of Medicine, New York) or rabbit polyclonal anti-CBP (1:5,000; Open Biosystems), anti-Rpl10 (1:1,000; B. Trumpower, Dartmouth Medical School, Hanover) and anti-Adh1 (1:50,000; obtained from the laboratory of C. De Virgilio, University of Fribourg) antibodies and secondary goat anti-mouse or goat anti-rabbit horseradish peroxidase-conjugated antibodies (Bio-Rad) were used. For detection of TAP-tagged proteins, peroxidase–anti-peroxidase soluble complex (1:20,000; Sigma) was used. Immobilized protein–antibody complexes were visualized by using enhanced chemiluminescence detection kits (Amersham ECL, GE Healthcare; PicoDetect, Applichem; WesternBright Sirius, Advansta).

### Protein purification for X-ray crystallography

For expression of the *C. thermophilum ct*Sqt1-(His)_6_ and *ct*Sqt1.52C-(His)_6_ proteins and the *ct*Rpl10(1–20)-(His)_6_/*ct*Sqt1.52C complex, *E. coli* BL21(DE3) cells, grown in lysogeny broth medium, were used. Expression of *S. cervevisiae* Sqt1.53C-(His)_6_ and Rpl10(1–20)-(His)_6_/Sqt1.53C was carried out in Rosetta(DE3) cells. BL21(DE3) cells expressing *ct*Sqt1.52C in Se-Met labelling conditions were grown in M9 medium containing 1 mM MgCl_2_ and 1 mM CaCl_2_, and supplemented with 125 mg lysine, 125 mg threonine, 125 mg phenylalanine, 50 mg valine, 50 mg leucine, 50 mg isoleucine, 5 g glucose and 50 mg Seleno-L-methionine per litre. Protein expression was induced with 1.8% (w/v) lactose, and cells were harvested after overnight growth at 30 °C and stored at −80 °C. Cells pellets were resuspended in 10 ml buffer A (20 mM HEPES (pH 8.0), 250 mM NaCl, 20 mM KCl, 20 mM MgCl_2_ and 40 mM imidazole) per gram of cells and lysed with a M-110L Microfluidizer (Microfluidics). The lysate was centrifuged at 20,000 r.p.m. for 20 min and the supernatant was applied onto a 5-ml HisTrap HP column (GE Healthcare) for Ni-NTA chromatography. The column was washed with five column volumes buffer A and proteins were eluted with buffer B (20 mM HEPES (pH 8.0), 250 mM NaCl, 20 mM KCl, 20 mM MgCl_2_ and 500 mM imidazole). Proteins were concentrated and further purified by size-exclusion chromatography using a HiLoad 26/60 Superdex 75 gel-filtration column in buffer C (20 mM HEPES (pH 8), 200 mM NaCl, 20 mM KCl and 20 mM MgCl_2_).

### Crystallization and structure determination

Purified proteins were concentrated to 25–30 mg ml^−1^ and crystallization screens were performed at 291 K by the sitting-drop vapor-diffusion method upon mixing equal volumes (0.5 μl) of protein solution and crystallization buffer with a reservoir volume of 100 μl. Full-length *ct*Sqt1 was crystallized in a condition containing 1.6 M (NH_4_)_2_SO_4_ and 100 mM MES (pH 6). Large brick-shaped crystals for native *ct*Sqt1.52C were obtained in 0.1 M Tris (pH 8.5) and 20% (v/v) ethanol after 24 h. Large plate-shaped crystals of *ct*Sqt1.52C-Se-Met for phase determination appeared after 4 days in 0.2 M di-ammonium tartrate and 20% (w/v) PEG3350. Needle-shaped crystals for *ct*Sqt1.52C/*ct*Rpl10(1–20) were obtained in 0.2 M sodium chloride, 0.1 M Na/K-phosphate (pH 6.2) and 40% PEG400 after 2 days. Large brick- and cube-shaped crystals were obtained for *Sc*Sqt1.53C in 95 mM Na-citrate (pH 5.6), 19% (v/v) isopropanol, 19% (w/v) PEG4000 and 5% glycerol after 1 month. Thick brick-shaped crystals were obtained for *Sc*Sqt1.53C/*Sc*Rpl10(1–20) in 0.2 M Ca-acetate, 0.1 M Na-cacodylate pH 6.5 and 40% (v/v) PEG600 after 4 days. Crystals were flash-frozen in liquid nitrogen after cryo-protection by transfer into cryo-solution containing mother liquor and 20% (v/v) glycerol. Diffraction data were measured under cryogenic conditions (100 K; Oxford Cryosystems Cryostream) at the European Synchrotron Radiation Facility (ESRF; Grenoble). Native crystals of *ct*Sqt1.52C and *ct*Sqt1.52C/*ct*Rpl10(1–20) were measured at ESRF beamline ID23-1. Se-Met-labelled *ct*Sqt1.52C, as well as native full-length *ct*Sqt1 and native *S. cerevisiae* Sqt1.53C and Sqt1.53C/Rpl10(1–20), were measured at ESRF beamline ID29. Data were processed with *iMOSFLM*^54^ and the *XDS* programme package^55^. In parallel to our efforts of determining the *ct*Sqt1.52C structure by Se-SAD, we performed a large number of molecular replacement trials for *ct*Sqt1 as implemented in *MOLREP*^56^. Potential solutions were verified and extended with *SHELXE*, as previously described^57^. The solution obtained with 3OW8 as a search model could be refined to an *R*_free_ of 45%, indicating a clear solution that could be further extended with *Buccaneer*^58^ and nearly completed with *ARP/wARP*^59^. The initial model for *ct*Sqt1.52C was obtained from a SAD Se-Met data set, with 5 of 6 Se-Met sites identified, using the AutoSol and AutoBuild programs of the *PHENIX* programme suite^60^. The obtained model was used for phasing of the native data set for *ct*Sqt1.52C at 1.5 Å resolution. The crystal structures of *ct*Sqt1.52C/*ct*Rpl10(1–20) (at 1.7 Å resolution), Sqt1.53C (at 2.0 Å resolution) and Sqt1.53C/Rpl10(1–20) (at 1.6 Å resolution) were obtained by molecular replacement using *Phaser*^61^ with *ct*Sqt1.52C and Sqt1.53C as search models, respectively. Model building and refinement of all structures were performed with the *PHENIX* programme suite^60^ and *Coot*^62^. Ramachandran statistics for the final model of *ct*Sqt1 molecular replacement (MR) show 97.6% of residues in most favourable regions, 2.4% in additionally allowed regions and 0% in disallowed regions. These statistical values for the final models of Sqt1.53C, *ct*Sqt1.52C, *ct*Sqt1.52C (Se-Met SAD), Sqt1.53C/L10(1–20) and *ct*Sqt1.52C/*ct*L10(1–20) were 95.8%/3%/1.2%, 96.5%/3.3%/0.2%, 96.2%/3.4%/0.4%, 96.9%/3.1%/0% and 96.7%/2.8%/0.5%, respectively. Figures were prepared in PyMOL (http://pymol.org/).

### Isothermal titration calorimetry

ITC experiments were performed on a MicroCal ITC 2000 instrument (GE Healthcare). The following peptides, corresponding to the N-terminal 19 (without N-terminal methionine) or 20 amino acids of *S. cerevisiae* and *C. thermophilum* Rpl10, were synthesized with a free acid group at the C terminus (peptides&elephants): Rpl10(1–20) NH2-MARRPARCYRYQKNKPYPKS-COOH, Rpl10(2–20) NH2-ARRPARCYRYQKNKPYPKS-COOH, *ct*Rpl10(1–20) NH2-MARRPARCYRYCKNKPYPKS-COOH and *ct*Rpl10(2–20) NH2-ARRPARCYRYCKNKPYPKS-COOH. Peptides (1 mg each) were dissolved in the appropriate volume of gel-filtration buffer (20 mM HEPES–NaOH (pH 7.5), 200 mM NaCl, 20 mM KCl and 20 mM MgCl_2_), which was used for the purification of *Sc*Sqt1.53C and *ct*Sqt1.52C, to obtain 1 mM stock solutions. Concentrations of proteins were determined by measuring the *A*_280_ using a NanoDrop Lite spectrophotometer (Thermo Scientific). For determination of the *C. thermophilum* Sqt1/L10-N binding, 200 μl of *ct*Sqt1.52C (10 μM) was added to the sample cell and 2 μl of 100 μM solutions of the peptides was titrated in at 25 °C. For determination of the *S. cerevisiae* Sqt1/L10-N binding, 200 μl of *Sc*Sqt1.53C (100 μM) was added to the sample cell and 2 μl of 1 mM solutions of the peptides were titrated in at 15 °C. ITC data were processed using the Origin ITC Software (OriginLab) and thermodynamic parameters were obtained by fitting the data to a One Set of Sites binding model.

### *In vivo* ribosomal protein solubility assay

The *sqt1* mutant cells, either containing empty vector or a centromeric plasmid expressing Sqt1 from the *ADH1* promoter, were grown in a volume of 100 ml to an OD_600_ of ∼0.7 and expression of C-terminally 2xHA-tagged Rpl10 was induced for 20 min from the *CUP1* promoter with 500 μM copper sulfate. After harvesting, cells were lysed with glass beads in a buffer containing 50 mM Tris-HCl (pH 7.5), 100 mM NaCl, 1.5 mM MgCl_2_, 5% glycerol and 0.1% NP-40, and cell extracts were centrifuged for 3 min at 3,000 r.p.m. Then, total cell extracts, 10 A_260_ units in a final volume of 500 μl, were subjected to centrifugation at 200,000*g* for 1 h. Pellets were resuspended in 100 μl lysis buffer and equal amounts of the total extracts (T), soluble extracts (S) and pellet fractions (P) were analysed by SDS–polyacrylamide gel electrophoresis and western blotting using an anti-HA antibody.

### Determination of co-translational capturing by qRT–PCR

Cells expressing Sqt1-TAP, NTAP-Rrb1, Syo1-FTpA and Yar1-TAP were grown at 30 °C in 400 ml YPD medium to an optical density (OD_600_) of 0.8, then cycloheximide was added to a final concentration of 0.2 mg ml^−1^ and the flasks were kept, with intermittent vigorous shaking, on ice for 5 min. Cells were harvested by centrifugation for 5 min at 4 °C at 4,000 r.p.m., washed once with 20 ml of ice-cold lysis buffer (100 mM NaCl, 50 mM Tris-HCl (pH 7.5), 1.5 mM MgCl_2_, 0.1% NP-40 and 5% glycerol) containing 1 mM PMSF and 0.2 mg ml^−1^ cycloheximide (lysis buffer-PC) and resuspended in 2 ml of lysis buffer-PC. Equal volumes of the resuspended cells were transferred into two 2.2 ml Eppendorf tubes, briefly centrifuged and resuspended in 600 μl of ice-cold lysis buffer-PC. To prevent RNA degradation, 4 μl of RiboLock (40 U μl^−1^; Fermentas) was added. Then glass beads, corresponding to 1/3 of the lysis buffer volume, were added and the tubes were vigorously vortexed for 10 × 30 s with 30-s intervals on ice. Cell extracts were transferred to a new tube and, to maximize the yield, 400 μl of ice-cold lysis buffer-PC was used to rinse the glass beads. Lysates were then clarified by centrifugation at 14,000 r.p.m. at 4 °C for 10 min and the two supernatants were combined in one 2.2 ml Eppendorf tube. A 50-μl aliquot (∼1/40 of total volume) was removed for the preparation of total RNA. Upon addition of 100 μl of IgG-sepharose beads, samples were incubated for 2 h on a turning wheel at 4 °C. Beads were then washed thrice with 1 ml of ice-cold lysis buffer-PC and twice with lysis buffer-PC containing additionally 1 mM dithiothreitol. After the last centrifugation, the wash buffer was completely removed and 500 μl ice-cold lysis buffer containing 1 mM dithiothreitol and 0.2 mg ml^−1^ cycloheximide, 5 μl RiboLock and 5 μl TEV protease (∼5 μg μl^−1^ stock) were added. TEV cleavage was carried out by overnight incubation at 4 °C on a turning wheel. Next morning, the IgG-sepharose beads were pelleted by centrifugation for 2 min at 1,800 r.p.m. and the supernatant (TEV eluate) was transferred to a 1.5-ml Eppendorf tube.

For RNA isolation from the TEV eluate aliquot (∼450 μl), the TEV eluate was adjusted to 10 mM EDTA (pH 8.0) and 0.5% SDS. Subsequently, two phenol:chloroform:isoamylalcohol (400 μl) extractions and one chloroform:isoamylalcohol (400 μl in 24:1 ratio) extraction were performed. After the last centrifugation for 5 min at 14,000 r.p.m., the aqueous phase (∼400 μl) was transferred to a new 1.5-ml Eppendorf tube and the RNA was precipitated by the addition of 40 μl of 3 M Na-acetate (pH 5.2) and 1 ml of 100% ethanol. After mixing by vortexing and incubation for 15 min at −20 °C, the tubes were centrifuged at 14,000 r.p.m. for 10 min at 4 °C. Pellets were washed once with 1 ml 70% ethanol and the tubes were centrifuged again at 14,000 r.p.m. for 10 min at 4 °C. The faintly visible pellets were briefly air-dried and then resuspended in 30 μl of diethylpyrocarbonate (DEPC)-treated dH_2_O. Total RNA was extracted from the cell extract aliquot (50 μl), upon addition of 250 μl of DEPC-treated dH_2_O and 100 μl of 4 × TES buffer (40 mM Tris-HCl (pH 7.5), 40 mM EDTA (pH 8) and 2% SDS), by two phenol:chloroform:isoamylalcohol extractions and one chloroform:isoamylalcohol extraction as described above. The precipitated RNA was finally dissolved in 50 μl of DEPC-treated dH_2_O. To remove any contaminating DNA before complementary DNA (cDNA) synthesis, the isolated total RNAs were treated with DNase using the DNA-free DNase Treatment & Removal Kit (Ambion). RNA concentrations (*A*_260_) were determined using a NanoDrop 1000 spectrophotometer (Thermo Scientific).

For cDNA synthesis by reverse transcription, the PrimeScript RT Reagent Kit (TaKaRa) was used according to the manufacturer's instructions. Reactions (40 μl) consisted of 8 μl of 5 × PrimeScript buffer, 2 μl of PrimeScript RT Enzyme Mix I, 2 μl of 50 μM Oligo dT Primer, 2 μl of 100 μM Random 6mers, 10 μl of RNA and 16 μl of RNase-free dH_2_O. The reaction mixture was incubated in a PCR machine for 15 min at 37 °C, heated up for 5 s to 85 °C and then cooled down to 4 °C.

For real-time qPCR, the Rotor-Gene SYBR Green PCR Kit (Qiagen) was used according to the manufacturer's instructions. Reactions (20 μl) consisted of 10 μl 2 × Rotor-Gene SYBR Green PCR Master Mix, 2 μl 10 μM forward and reverse primer, 1 μl cDNA and 5 μl RNase-free dH_2_O. Real-time qPCRs were run in the Rotor-Gene Q real-time PCR cycler (Qiagen). The following real-time qPCR programme was used: 5 min at 95 °C (initial denaturation and activation of HotStarTaq *Plus* DNA polymerase), 5 s at 95 °C (denaturation), 10 s at 60 °C (annealing), 8 s at 72 °C (elongation and fluorescence data collection), 45 cycles. Genomic DNA of W303 (13, 1.3 and 0.13 ng, which correspond to ∼1,000,000, 100,000 and 10,000 gene per mRNA copies, respectively) was used as copy-number standard. The following oligonucleotide pairs were used for the specific amplification of DNA fragments, corresponding to the *RPL3*, *RPL5*, *RPL10* and *RPS3* mRNAs, from the input cDNAs: RPL3-I-forward 5′-ACTCCACCAGTTGTCGTTGTTGGT-3′ and RPL3-I-reverse 5′-TGTTCAGCCCAGACGGTGGTC-3′ (amplicon size 86 base pairs (bp)), RPL5-I-forward 5′-TAGCTGCTGCCTACTCCCACGA-3′ and RPL5-I-reverse 5′-GCAGCAGCCCAGTTGGTCAAA-3′ (amplicon size 70 bp), RPL10-I-forward 5′-TGTCTTGTGCCGGTGCGGAT-3′ and RPL10-I-reverse 5′-TGTCGACACGAGCGGCCAAA-3′ (amplicon size 84 bp), and RPS3-I-forward 5′-GCTGCTTACGGTGTCGTCAGAT-3′ and RPS3-I-reverse 5′-AGCCTTAGCTCTGGCAGCTCTT-3′ (amplicon size 96 bp). The yEGFP mRNA was amplified with the oligonucleotide pair yEGFP-II-forward 5′-TCACTGGTGTTGTCCCAATT-3′ and yEGFP-II-reverse 5′-ACCTTCACCGGAGACAGAAA-3′ (amplicon size 77 bp). Oligonucleotides were designed by using the Primer3 software (http://bioinfo.ut.ee/primer3-0.4.0/).

Real-time qRT–PCRs were performed in triplicate with all four oligonucleotide pairs using the same cDNA, derived from the total RNAs or the RNAs extracted from the TEV eluates of each of the four chaperone expressing strains. The threshold cycle (*C*_t_) was determined for each qPCR and the Δ*C*_t_ between the average of the triplicate ‘total RNA' qPCRs and each of the triplicate ‘TEV eluate' qPCRs were calculated. These values were then expressed as fold difference in template abundance between TEV eluate and total extract (ratio TEV/total) for each of the four mRNAs (*RPL3*, *RPL5*, *RPL10* and *RPS3*). In each case, the average fold difference in TEV/total ratio of the specifically associated mRNA (for example, *RPL10* mRNA in Sqt1-TAP derived TEV eluate and total extract) was set to 1 and, accordingly, the normalized TEV/total ratio values for the remaining three mRNAs were determined. Thus, the derived values (average and standard deviation) represent the relative enrichment of the four ribosomal protein encoding mRNAs (*RPL3*, *RPL5*, *RPL10* and *RPS3*) in each of the four chaperone purifications (NTAP-Rrb1, Syo1-FTpA, Sqt1-TAP and Yar1-TAP).

## Additional information

**Accession codes:** Protein Data Bank: atomic coordinates and structure factors for Sqt1.53C, Sqt1.53C/Rpl10(1–20), *ct*Sqt1, *ct*Sqt1.52C and *ct*Sqt1.52C/*ct*Rpl10(1–20) have been deposited under accession codes 4ZOV, 4ZOX, 4ZN4, 4ZOY and 4ZOZ, respectively.

**How to cite this article:** Pausch, P. *et al.* Co-translational capturing of nascent ribosomal proteins by their dedicated chaperones. *Nat. Commun.* 6:7494 doi: 10.1038/ncomms8494 (2015).

## Supplementary Material

Supplementary Information Supplementary Figures 1-11, Supplementary Tables 1-3 and Supplementary References

## Figures and Tables

**Table 1:** Data collection, phasing and refinement statistics for Sqt1.

	***Sc*****Sqt1.53C**	***Ct*****Sqt1.52C**	***Ct*****Sqt1.52CSe-Met SAD**	***Ct*****Sqt1MR**
*Data collection*				
Space group	P2_1_	P2_1_2_1_2_1_	C2	C2
Cell dimensions				
*a*, *b*, *c* (Å)	53.79	67.44	111.44	111.56
	75.17	73.02	93.68	94.82
	101.25	113.89	186.85	94.95
*α*, *β*, *γ* (°)	90.00	90.00	90.00	90.00
	104.42	90.00	104.20	109.90
	90.00	90.00	90.00	90.00
			Peak	
Wavelength			1.0332	
Resolution (Å)*	49.03–2.00 (2.11–2.00)	37.38–1.50 (1.58–1.50)	46.84–2.30 (2.38–2.30)	47.41–1.94 (1.99–1.94)
*R*_merge_	0.063 (0.091)	0.076 (0.458)	0.141 (0.732)	0.055 (0.727)
*I*/*σI*	16.5 (11.4)	11.2 (3.1)	15.15 (3.7)	14.6 (1.9)
Completeness (%)	98.5 (98.1)	98.7 (98.7)	99.8 (99.8)	98.6 (96.9)
Redundancy	4.4 (4.3)	5.9 (5.8)	13.8 (13.9)	4.8 (4.1)
				
*Refinement*				
Resolution (Å)	42.57–2.00	37.38–1.50	46.84–2.30	47.41–1.94
No. of reflections	51,857	89,196	82,688	67,765
*R*_work_/*R*_free_	18.7/22.3	18.4/20.1	21.7/26.8	17.13/20.52
No. of atoms	6,367	3,569	12,312	6,359
Protein	5,727	3,009	11,774	6,037
Ligand/ion	0	0	0	17
Water	640	560	538	305
B-factors	28.30	24.30	26.30	38.70
Protein	27.50	22.00	26.20	38.60
Ligand/ion	—	—	—	39.00
Water	35.70	36.40	28.50	40.00
R.m.s.d.'s				
Bond lengths (Å)	0.008	0.007	0.009	0.009
Bond angles (°)	1.2	1.1	1.2	1.2

R.m.s.d., root mean squared deviation; SAD, single-anomalous dispersion.

The following amino acid residues could not be resolved due to lacking electron density:

*Sc*Sqt1.53C: chain A: none/chain B: E302-Q307

*Ct*Sqt1.52C: A52-L53, A106-N126, S224-S227, S332-H365 and G467-P488

*Ct*Sqt1: Chain A: M1-I51, A105-N126, A223-S227, P334-Q366 and Q473-A486 /

Chain B: M1-A52, G107-N126, A223-D225, P334-Q366 and M472-A486

^*^Values in parentheses are for highest-resolution shell.

**Table 2:** Data collection and refinement statistics for Sqt1/L10-N.

	***Sc*****Sqt1.53C/*****Sc*****L10(1–20)**	***Ct*****Sqt1.52C/*****Ct*****L10(1–20)**
*Data collection*		
Space group	C2	P1
Cell dimensions		
*a*, *b*, *c* (Å)	74.27	47.50
	89.25	67.18
	53.56	72.82
*α*, *β*, *γ* (°)	90.00	89.92
	100.28	108.92
	90.00	92.03
Resolution (Å)*	44.63–1.60 (1.69–1.60)	48.33–1.70 (1.79–1.70)
*R*_merge_	0.052 (0.092)	0.054 (0.415)
*I*/*σΙ*	18.5 (11.2)	13.6 (3.0)
Completeness (%)	99.4 (98.6)	93.7 (91.8)
Redundancy	4.0 (3.9)	3.7 (3.7)
		
*Refinement*		
Resolution (Å)	44.63–1.60	48.33–1.70
No. of reflections	44,971	87,711
*R*_work_/*R*_free_	14.6/18.1	19.1/21.6
No. of atoms	3,574	6,591
Protein	3,015	6,026
Ligand/ion	0	0
Water	559	565
B-factors	14.20	26.10
Protein	12.30	25.40
Ligand/ion	—	—
Water	24.30	33.80
R.m.s.d.'s		
Bond lengths (Å)	0.007	0.007
Bond angles (°)	1.1	1.1

R.m.s.d., root mean squared deviation

The following amino acid residues could not be resolved due to lacking electron density:

*Sc*Sqt1.53C/*Sc*L10(1–20): none/M1 and P16-S20

*Ct*Sqt1.52C/*Ct*L10(1–20): A52-L53, A104-T127, S332-Q366 and T463-S489/M1 and N14-S20

^*^Values in parentheses are for highest-resolution shell.
