# Supplementary material for: Co-translational capturing of nascent ribosomal proteins by their dedicated chaperones
Source: Nat Commun. 2015 Jun 26;6:7494. doi: 10.1038/ncomms8494 (PMC4491177; doi:10.1038/ncomms8494)
Supplement: Supplementary Information — Supplementary Figures 1-11, Supplementary Tables 1-3 and Supplementary References [file ncomms8494-s1.pdf]

Supplementary Information

Co-translational capturing of nascent ribosomal proteins by their dedicated chaperones

Patrick Pausch[1,4], Ujjwala Singh[2,4], Yasar Luqman Ahmed[3,4], Benjamin Pillet[2,4], Guillaume Murat[2], Florian Altegoer[1], Gunter Stier[3], Matthias Thoms[3], Ed Hurt[3], Irmgard Sinning[3], Gert Bange[1,5], and Dieter Kressler[2,5]

[1]LOEWE Center for Synthetic Microbiology (SYNMIKRO) and Department of Chemistry, Philipps-University Marburg, Hans-Meerwein-Straße, D-35043 Marburg, Germany
[2]Unit of Biochemistry, Department of Biology, University of Fribourg, Chemin du Musée 10, CH-1700 Fribourg, Switzerland
[3]Heidelberg University Biochemistry Center (BZH), Im Neuenheimer Feld 328, D-61920 Heidelberg, Germany

[4]These authors contributed equally to this work
[5]Correspondence and requests for materials should be addressed to G.B. (email: gert.bange@synmikro.uni-marburg.de) and D.K. (email: dieter.kressler@unifr.ch).

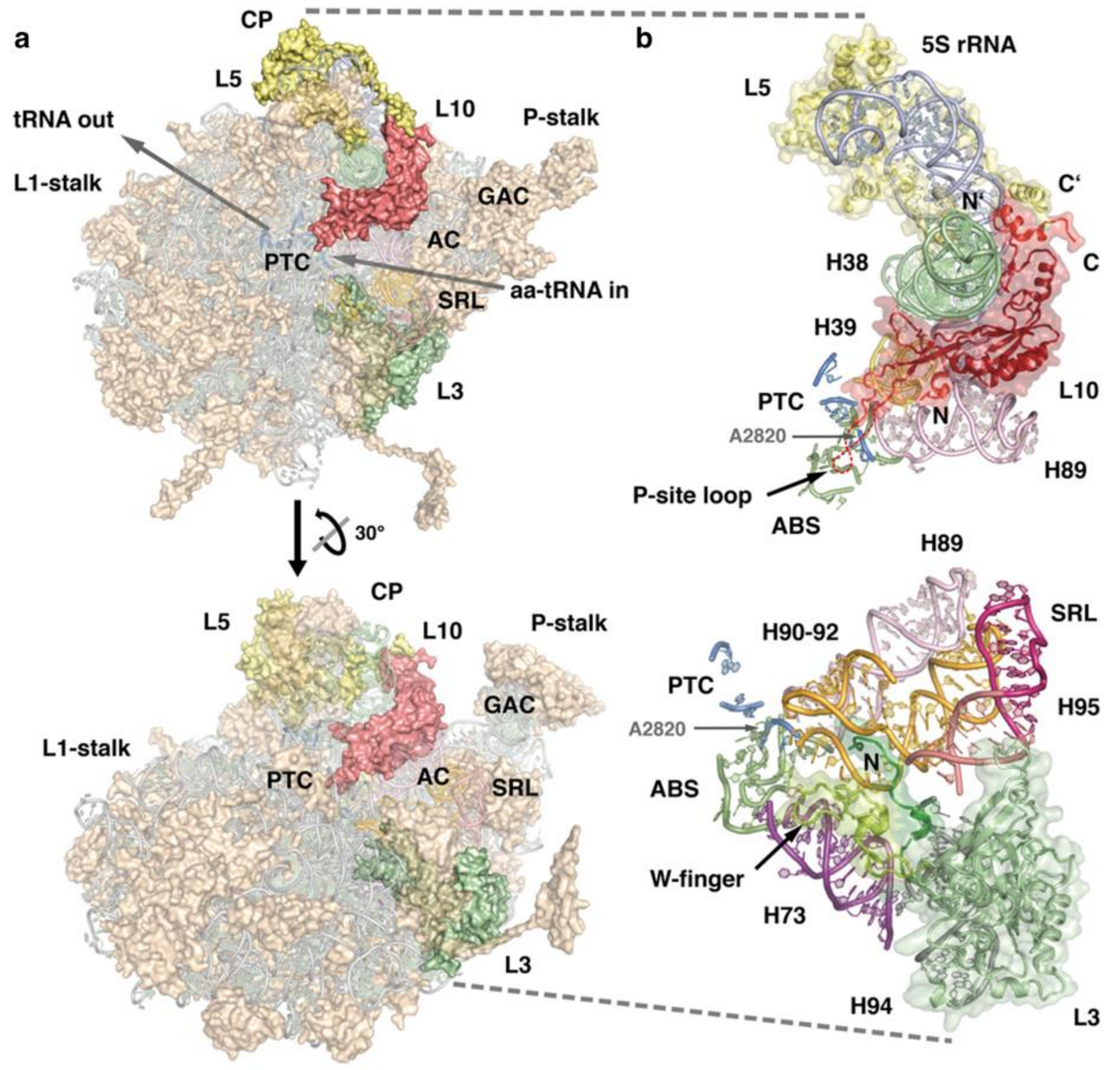

Supplementary Figure 1 Location of the r-proteins Rpl10 and Rpl3 within 60S r-subunits. **(a)** For visualization of the position of Rpl10 (L10), the 60S r-subunit is shown from the interface side in its classical crown view (upper part). To better visualize the position of Rpl3 (L3), the 60S r-subunit has been turned for 30º along the indicated axis (lower part). For orientation purposes, the approximate positions of the following 60S subunit landmarks are indicated: L1-stalk, central protuberance (CP), P-stalk, peptidyl transferase center (PTC), sarcin-ricin loop (SRL), GTPase associated center (GAC), and accommodation corridor (AC). The entry and exit path of the charged (aa-tRNA) and uncharged tRNAs is also indicated. Rpl10 is shown in deep salmon, Rpl3 in pale green, and Rpl5 (L5) in pale yellow. The rRNA and r-protein moieties that are not relevant for this study are transparently colored in grey and wheat, respectively. **(b)** Close-up views highlighting the 60S r-subunit environment of Rpl10 and Rpl3. Rpl10 is sandwiched between rRNA helices H38 and H89

and its N-terminal residues interlock into H89 (upper part). Dashed lines indicate the position of the internal P-site loop of Rpl10, which is disordered in the 60S structure. Rpl5 (L5) is shown in pale yellow, the 5S rRNA in light blue, H38 in pale green, H39 in yellow orange, and H89 in light pink. The N and C termini of Rpl10 (N and C) and Rpl5 (N' and C') are indicated. Important PTC residues, with the position of base A2820 being indicated by a grey arrow, and the anisomycin binding site (ABS) are shown in marine blue and smudge green, respectively. The globular domain of Rpl3 (shown in split pea green) is located on the solvent-side surface of the 60S subunit in close proximity of the SRL (lower part). The N-terminal extension (shown in forest green) and the internal W-finger loop (shown in limon green) emanate from the globular domain deep into the central core of the 60S r-subunit. The position of the N-terminus of Rpl3 is indicated. H73 is shown in purple, H89 in light pink, H90-92 in bright orange, H94 in grey, H95 in light magenta, and the SRL in warm pink. The shown structures were generated in PyMOL using the PyMOL session file of the *S. cerevisiae* 80S ribosome (based on PDB 3U5F, 3U5G, 3U5H, and 3U5I)[1] provided on the Ban laboratory web site (http://www.mol.biol.ethz.ch/groups/ban_group/nomenclature).

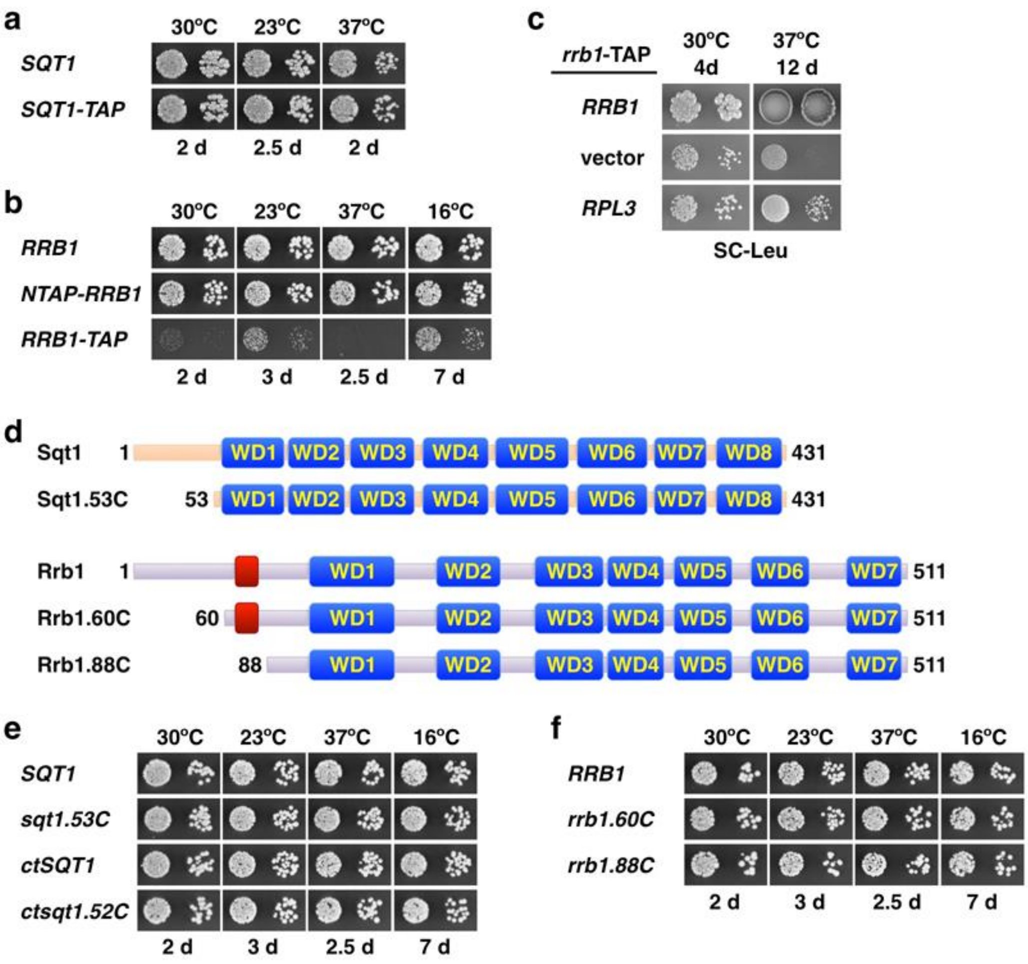

Supplementary Figure 2 The *SQT1*-TAP and NTAP-*RRB1* constructs are fully functional and the WD-repeat β-propeller domain of Sqt1 and Rrb1 is sufficient to sustain wild-type growth. **(a)** Growth phenotype of cells expressing Sqt1-TAP from the genomic locus. Cells of an isogenic wild-type strain (*SQT1*) and the *SQT1*-TAP strain were spotted in 10-fold serial dilution steps onto YPD plates, which were incubated for the indicated times at 30ºC, 23ºC and 37ºC. **(b)** Growth phenotype of cells expressing NTAP-Rrb1 from a monocopy plasmid under the control of the cognate *RRB1* promoter in an *rrb1* null strain. The *RRB1* shuffle strain was transformed with YCplac111-based plasmids harbouring the *RRB1* wild-type gene or the NTAP-*RRB1* construct. After plasmid shuffling on 5-FOA-containing plates, cells were restreaked on YPD plates and then spotted in 10-fold serial dilution steps onto YPD plates, which were incubated for the indicated times at 30ºC, 23ºC, 37ºC and 16ºC. For comparison cells expressing C-terminally TAP-tagged Rrb1 (*RRB1*-TAP) from the genomic locus were also spotted. **(c)** Overexpression of Rpl3 weakly suppresses the slow-growth phenotype of *rrb1*-TAP mutant cells. The *RRB1*-TAP strain was transformed with empty vector or monocopy plasmids harbouring either *RRB1* or *RPL3*. Cells were spotted in 10-fold

serial dilution steps onto SC-Leu plates, which were incubated for 4 d at 30ºC or for 12 d at 37ºC. **(d)** Schematic representation of Sqt1 and Rrb1. The WD repeats of Sqt1 were positioned according to the crystal structure reported in this study. The N-terminal deletion variant Sqt1.53C lacks amino acids 1-52 and starts with amino acid 53. Rrb1 is predicted to contain a 7-bladed WD-repeat β-propeller domain that is preceded by a predicted α-helix and an N-terminal extension, which is devoid of any clearly assignable secondary structure elements. The secondary structure of Rrb1 was predicted with the PSIPRED v3.3 prediction method available at the PSIPRED website interface[2]. The number and position of Rrb1's WD-repeats were assigned according to the output of the WDSP program[3], sequence alignments, and secondary structure prediction. The N-terminal deletion variants Rrb1.60C (deletion of amino acids 2-59) and Rrb1.88C (deletion of amino acids 2-87) start with amino acid 60 and 88, respectively. **(e)** The WD-repeat β-propeller domain of Sqt1 is sufficient to confer full functionality and the *C. thermophilum* ortholog *ct*Sqt1 can functionally replace Sqt1. Plasmid-borne wild-type *SQT1* or *sqt1.53C*, expressed from the cognate promoter, and *ctSQT1* or *ctsqt1.52C*, under the control of the *ADH1* promoter, were transformed into the *SQT1* shuffle strain. After plasmid shuffling on 5-FOA-containing plates, cells were restreaked on YPD plates and then spotted in 10-fold serial dilution steps onto YPD plates, which were incubated for the indicated times at 30ºC, 23ºC, 37ºC and 16ºC. **(f)** The WD-repeat β-propeller domain of Rrb1 is sufficient to confer full functionality. Plasmid-borne wild-type *RRB1* or the *rrb1.53C* and *rrb1.88C* N-terminal deletion constructs, under the control of the *ADH1* promoter, were transformed into the *RRB1* shuffle strain. After plasmid shuffling on 5-FOA-containing plates, cells were restreaked on YPD plates and then spotted in 10-fold serial dilution steps onto YPD plates, which were incubated for the indicated times at 30ºC, 23ºC, 37ºC and 16ºC.

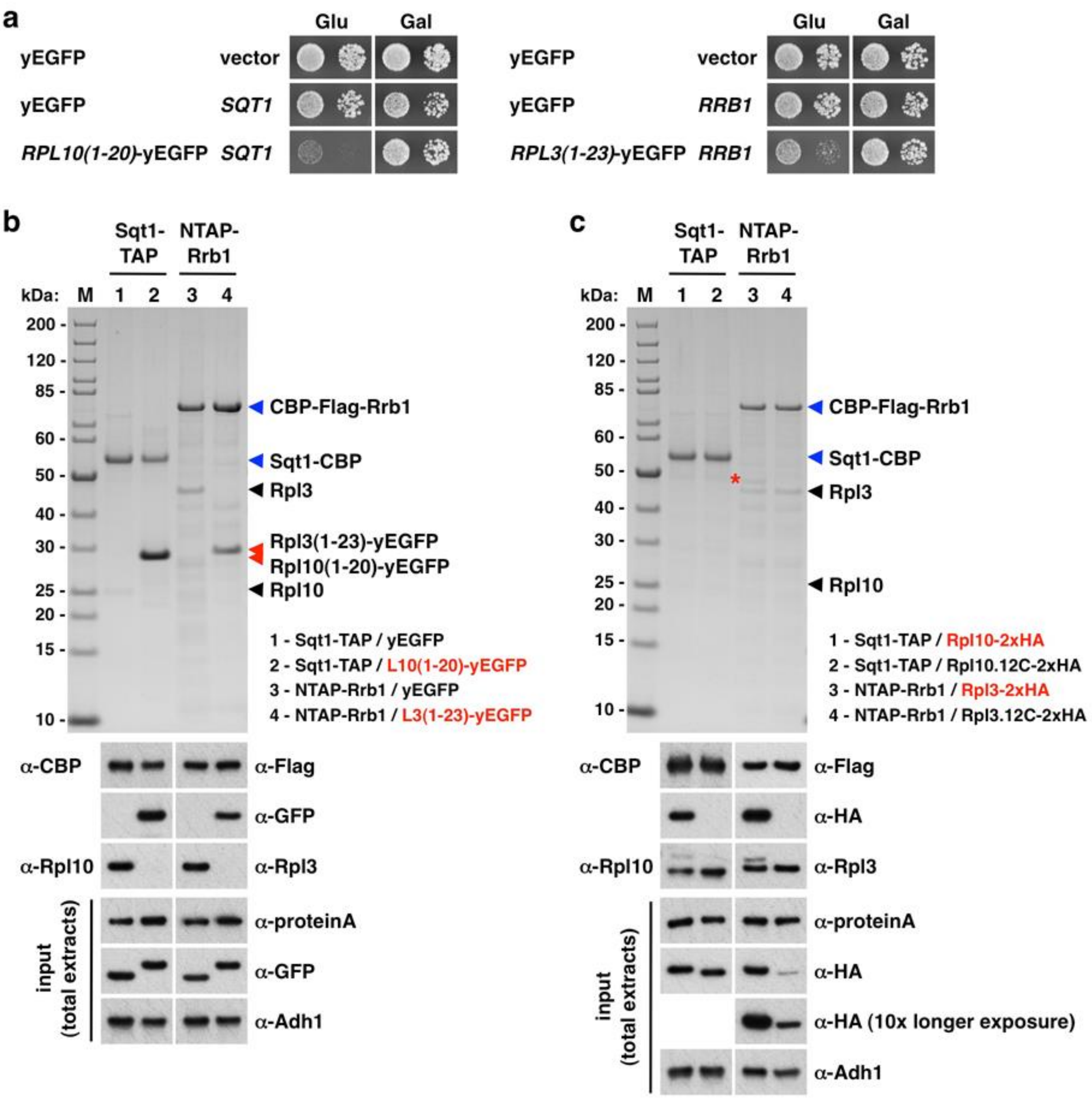

Supplementary Figure 3 The N-terminal residues of Rpl10 and Rpl3 are sufficient and required for the interaction with Sqt1 and Rrb1, respectively. **(a)** The negative growth effect associated with L10-N- and L3-N-yEGFP expression is suppressed by overexpression of Sqt1 and Rrb1, respectively. The wild-type strain YDK11-5A was co-transformed with plasmids expressing yEGFP, Rpl10(1-20)-yEGFP or Rpl3(1-23)-yEGFP from the cognate *RPL10* or *RPL3* promoter, respectively, and empty vector or plasmids expressing Sqt1 or Rrb1 from the inducible *GAL1-10* promoter. Transformants were first selected and then restreaked on SGal-Leu-Trp plates. Cells were spotted in 10-fold serial dilution steps onto SC-Leu-Trp (Glucose, Glu) and SGal-Leu-Trp (Galactose, Gal) plates, which were incubated for 3 d or 4 d at 30ºC, respectively. **(b)** The N-terminal residues of Rpl10 and Rpl3 are sufficient to mediate interaction with their chaperones *in vivo*. Tandem-affinity purification of C-terminally TAP-tagged Sqt1 (Sqt1-TAP, lanes 1 and 2) and N-terminally TAP-tagged Rrb1 (NTAP-Rrb1,

lanes 3 and 4) from cells where expression of the yEGFP control protein (lanes 1 and 3) or the Rpl10(1-20)-yEGFP (lane 2) and Rpl3(1-23)-yEGFP (lane 4) fusion proteins has been induced from the *CUP1* promoter for 30 min with 500 μM copper sulfate. Final EGTA eluates were analyzed by SDS-PAGE and Coomassie staining (top) or by Western blotting using anti-CBP, anti-Flag, anti-GFP, anti-Rpl10, and anti-Rpl3 antibodies (bottom). Total cell extracts (input) were analyzed by Western blotting using anti-proteinA, anti-GFP, and anti-Adh1 (loading control) antibodies. M, molecular weight standard. **(c)** The N-terminal residues of Rpl10 and Rpl3 are required for the interaction with Sqt1 and Rrb1 *in vivo*. Tandem-affinity purification of C-terminally TAP-tagged Sqt1 (Sqt1-TAP, lanes 1 and 2) and N-terminally TAP-tagged Rrb1 (NTAP-Rrb1, lanes 3 and 4) from cells where expression of Rpl10-2xHA (lane 1), Rpl10.12C-2xHA (lane 2), Rpl3-2xHA (lane 3), and Rpl3.12C-2xHA (lane 4) has been induced from the *CUP1* promoter for 30 min with 500 μM copper sulfate. Final EGTA eluates were analyzed by SDS-PAGE and Coomassie staining (top) or by Western blotting using anti-CBP, anti-Flag, anti-HA, anti-Rpl10, and anti-Rpl3 antibodies (bottom). The red asterisk indicates the Rpl3-2xHA band in lane 3 of the Coomassie-stained SDS-PAGE. Total cell extracts (input) were analyzed by Western blotting using anti-proteinA, anti-HA, and anti-Adh1 (loading control) antibodies. Note that the co-purification of Rpl10 and Rpl3 with the Sqt1-TAP and NTAP-Rrb1 bait proteins, respectively, is less efficient when cells are grown in synthetic medium.

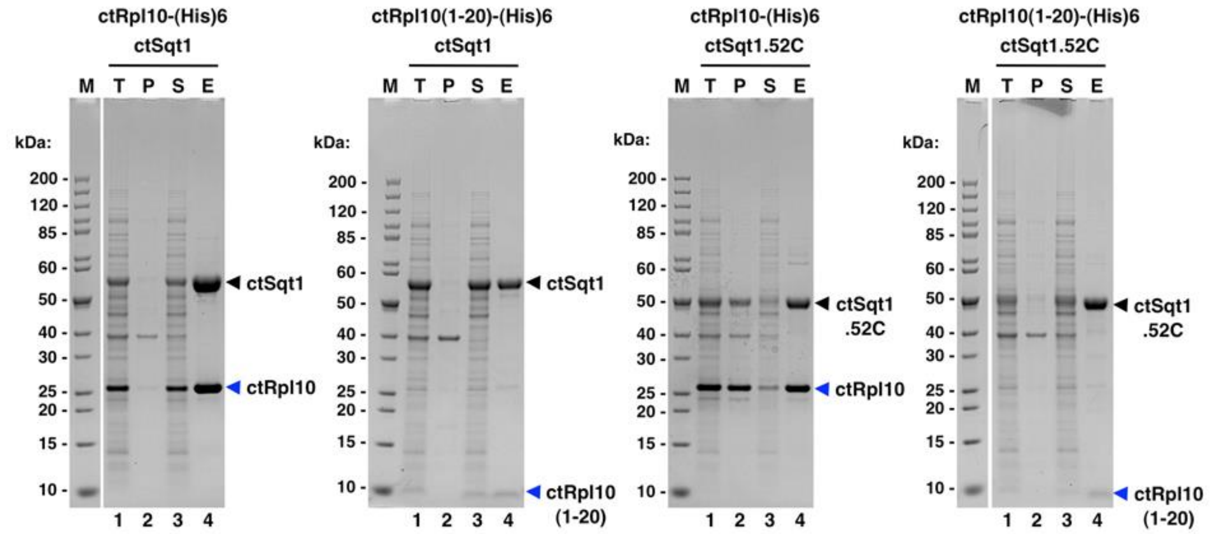

Supplementary Figure 4 The N-terminal 20 residues of *ct*Rpl10 interact with the WD-repeat β-propeller domain of *ct*Sqt1. *In vitro* binding assays between *ct*Rpl10 and *ct*Sqt1. The indicated C-terminally $(His)_6$-tagged *ct*Rpl10 and non-tagged *ct*Sqt1 variants were co-expressed in *E. coli*, purified *via* Ni-affinity purification and proteins were revealed by SDS-PAGE and Coomassie staining. T, total extract (lane 1); P, pellet fraction (insoluble proteins, lane 2); S, soluble extract (lane 3); E, imidazole eluate (lane 4); M, molecular weight standard. The bands highlighted by blue arrowheads correspond to the different *ct*Rpl10 variants used as baits for the purifications. Black arrowheads indicate the position of *ct*Sqt1 and *ct*Sqt1.52C.

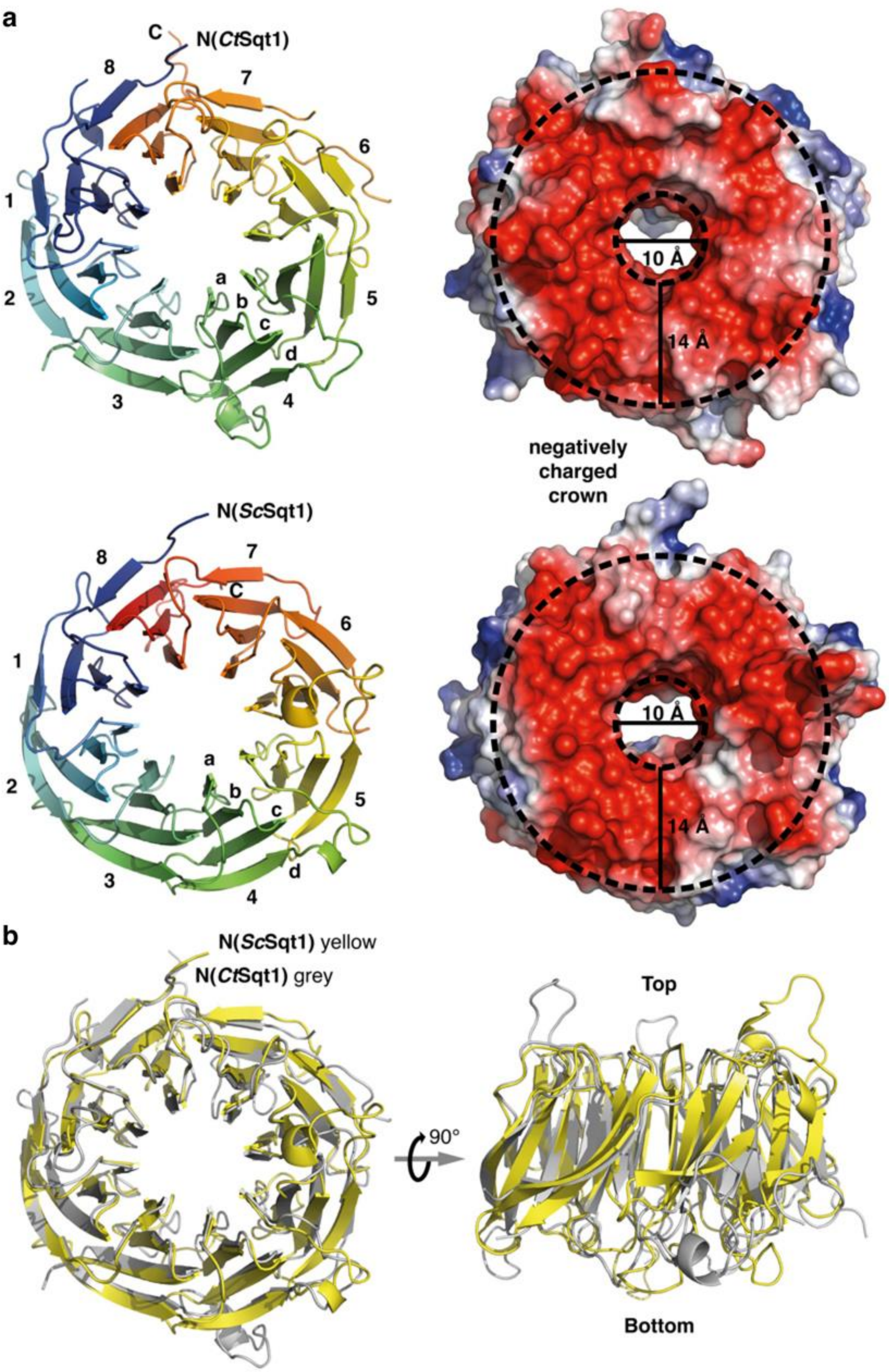

Supplementary Figure 5 Crystal structures of the eight-bladed WD-repeat β-propeller domain of Sqt1 from *S. cerevisiae* and *C. thermophilum*. **(a)** Crystal structures of *Ct*Sqt1.52C (upper panel) and *Sc*Sqt1.53C (lower panel). Cartoon representation showing Sqt1 in rainbow

colors from N- to C-terminus (left panels). The eight-bladed WD-repeat β-propeller is shown in its top view. Assignment of the top and bottom surface as well as numbering of the propeller blades (1-8) and labeling of the β-strands within each blade (a to d) is according to the conventional definition for WD-repeat β-propellers. N- and C-termini are indicated. The following *Ct*Sqt1.52C residues, which are located in the loops connecting β-strands 1c-1d, 3c-3d, 5c-5d, and 7c-7d, could not be assigned due to lacking electron density: Ala106-Asn126, Ser224-Ser227, Ser332-His365, and Gly467-Pro488. Analysis of the electrostatic properties reveals that the top surface of Sqt1 contains a cluster of negatively charged residues, indicated as 'negatively charged crown' (right panels). **(b)** The WD-repeat β-propeller domains of *Sc*Sqt1 and *Ct*Sqt1 share a similar overall architecture. The crystal structures of *Sc*Sqt1.53C (yellow) and *Ct*Sqt1.52C (grey) were aligned in PyMOL and are shown as cartoon representations in their top (left panel) and side views (right panel).

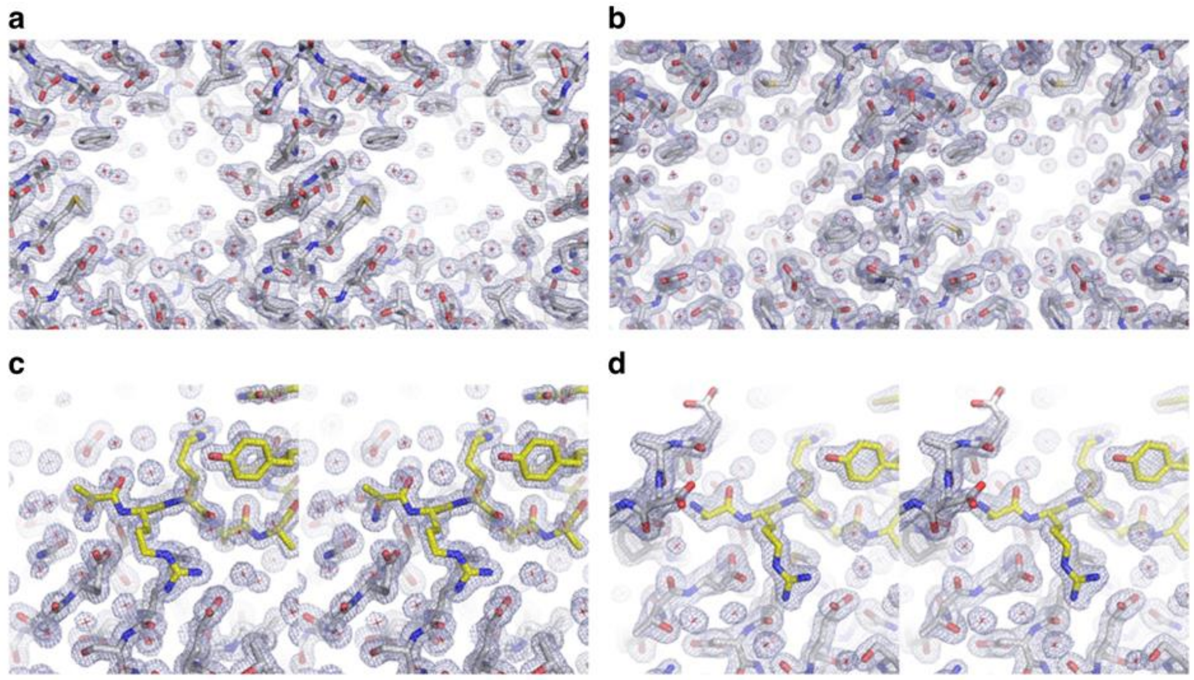

Supplementary Figure 6 Stereo images of the electron density maps of portions of the reported structures. **(a)** Stereo view of close-up image of central channel, viewed from the top surface, of *Sc*Sqt1.53C. **(b)** Stereo view of close-up image of central channel, viewed from the top surface, of *Ct*Sqt1.52C. **(c)** Stereo view of the sloped top surface of *Sc*Sqt1.53C (grey, coloured by element) with bound *Sc*L10-N (yellow, coloured by element), focusing on the first N-terminal residues of *Sc*L10. **(d)** Stereo view of the sloped top surface of *Ct*Sqt1.52C (grey, coloured by element) with bound *Ct*L10-N (yellow, coloured by element), focusing on the first N-terminal residues of *Ct*L10. The $2F_{obs}$-F_{calc} electron density maps (light blue mesh), contoured at 1.0 σ after final refinement, are shown.

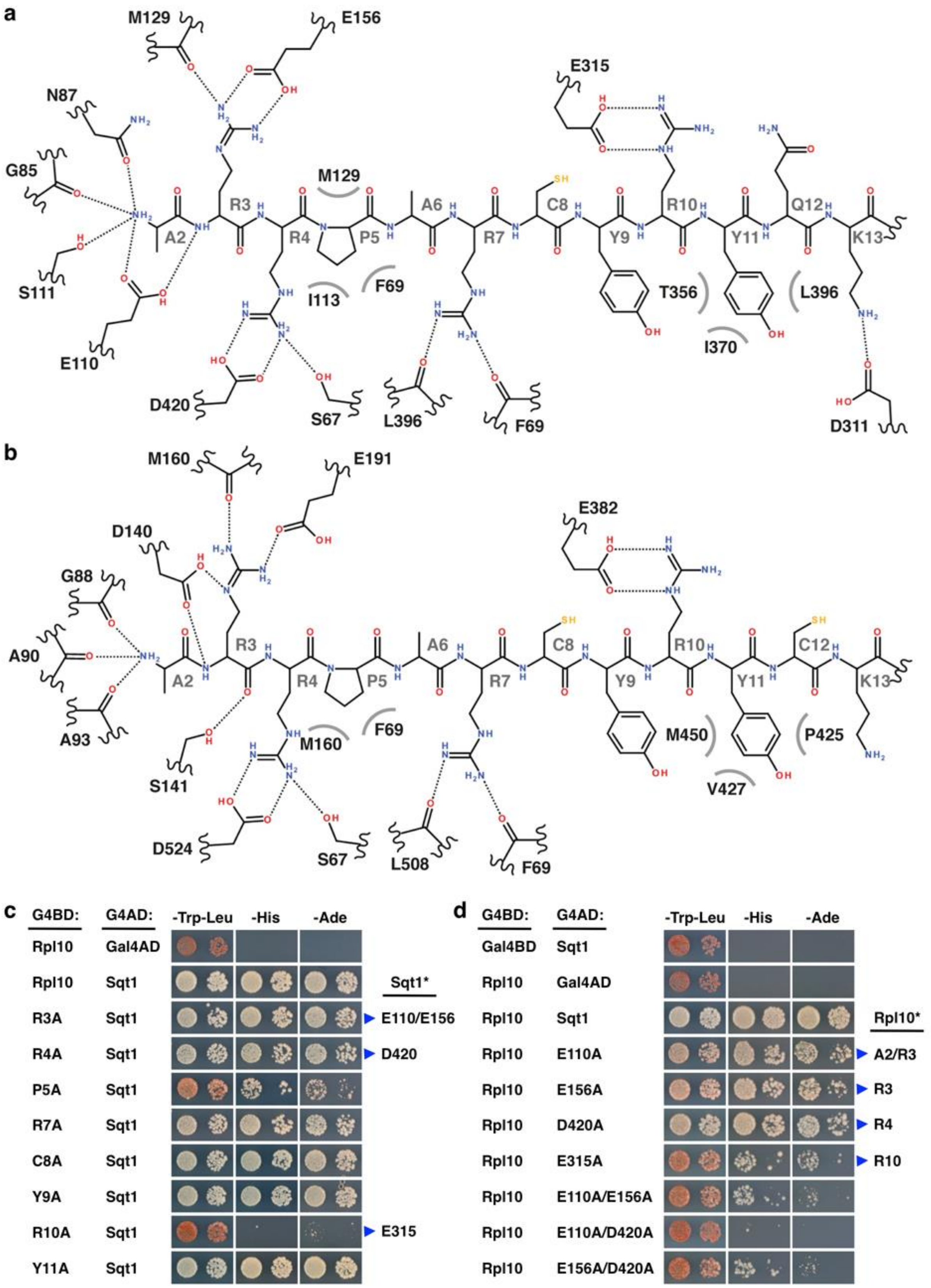

Supplementary Figure 7 Mutational analysis of the interaction between Sqt1 and L10-N. **(a)** Representation of the mode of L10-N recognition by *Sc*Sqt1. The backbone and side chains of *Sc*L10-N (residues 2-13) are shown as an elongated peptide. L10-N residues are labeled in

grey (*e.g.*: A2 for Ala2). The Sqt1 residues that form interactions, either *via* their side chains or main chain carbonyls, with the L10-N peptide are indicated. Dotted lines indicate ionic interactions or hydrogen bonds and grey, curved lines hydrophobic interactions. The interaction representation was created with Accelrys Draw 4.1. **(b)** Representation of the mode of *ct*L10-N recognition by *ct*Sqt1. The backbone and side chains of *ct*L10-N (residues 2-13) are shown as an elongated peptide. The *ct*L10-N residues are labeled in grey (*e.g.*: A2 for Ala2). The *ct*Sqt1 residues that form interactions, either *via* their side chains or main chain carbonyls, with the *ct*L10-N peptide are indicated. Dotted lines indicate ionic interactions or hydrogen bonds and grey, curved lines hydrophobic interactions. **(c)** Y2H interaction between Sqt1 and Rpl10 variants harboring mutations within the N-terminal residues. The residues mutated in Rpl10 (*e.g.*: R3A for Arg3 to alanine), as well as the Sqt1 residues they are contacting (blue arrowheads, Sqt1*), are indicated. **(d)** Y2H interaction between Rpl10 and mutant Sqt1 variants. The residues mutated in Sqt1 (*e.g.*: E110A for Glu110 to alanine), as well as the L10-N residues they are contacting (blue arrowheads, Rpl10*), are indicated. Single-letter abbreviations for the amino acid residues are as follows: A, Ala; C, Cys; D, Asp; E, Glu; P, Pro; R, Arg; and Y, Tyr.

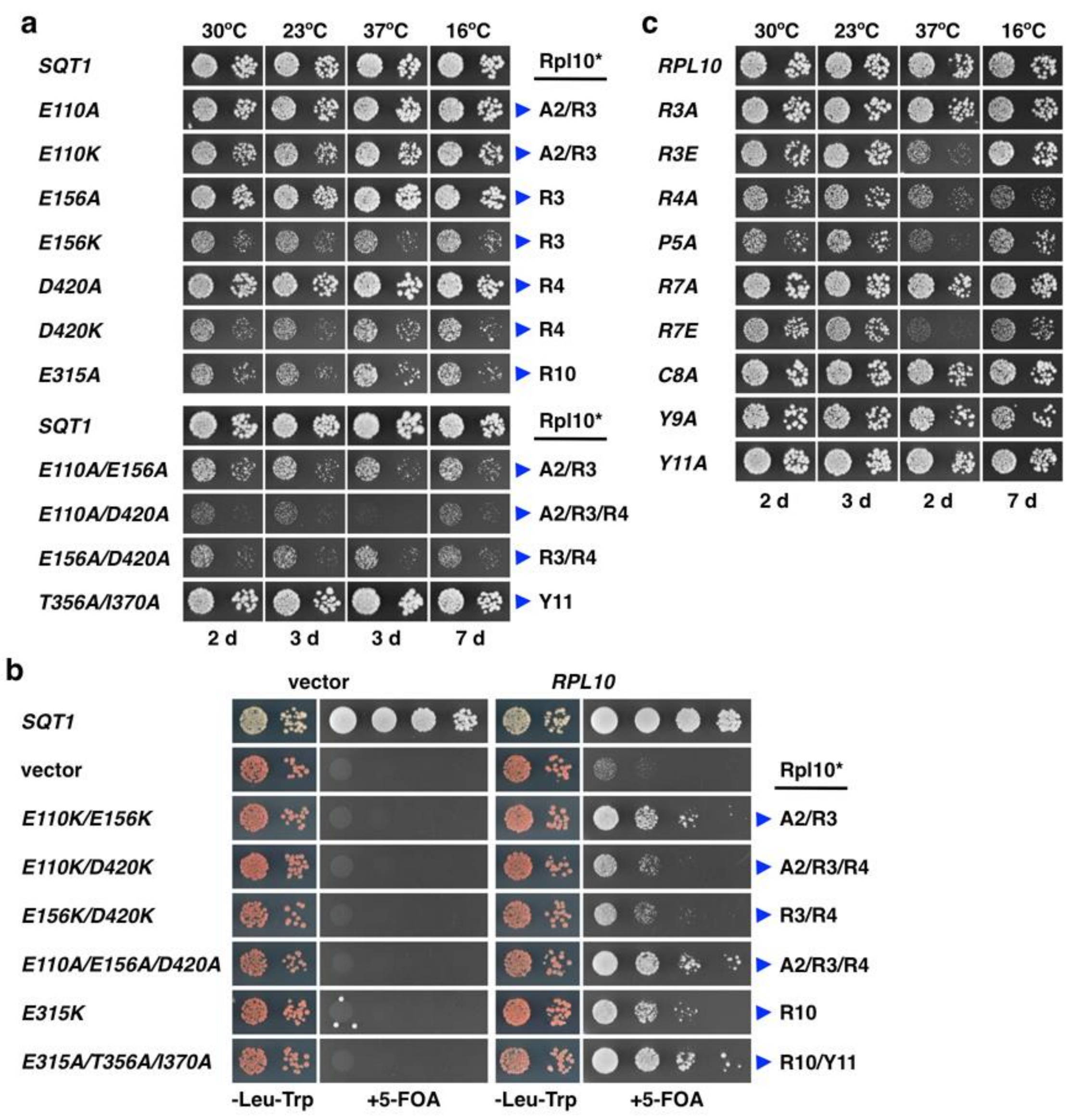

Supplementary Figure 8 Overexpression of Rpl10 bypasses the requirement for the essential Sqt1. **(a)** *In vivo* phenotypes of cells expressing viable Sqt1 variants that affect the interaction with Rpl10. YCplac111-based plasmids harbouring the *SQT1* wild-type gene or the indicated *sqt1* alleles, under the control of the cognate promoter, were transformed into the *SQT1* shuffle strain. After plasmid shuffling on 5-FOA-containing plates, cells were restreaked on YPD plates and then spotted in 10-fold serial dilution steps onto YPD plates, which were incubated for the indicated times at 30ºC, 23ºC, 37ºC and 16ºC. The residues mutated in Sqt1, as well as the L10-N residues they are contacting (blue arrowheads, Rpl10*), are indicated. **(b)** Overexpression of Rpl10 confers weak growth to cells expressing lethal Sqt1 variants and even rescues the absence of Sqt1. The *SQT1* shuffle strain was co-transformed with YCplac111-based plasmids harboring the *SQT1* wild-type gene or the indicated *sqt1* alleles and the multicopy plasmid YEplac112-*RPL10* or the empty control

vector. These constructs express *SQT1* and *RPL10* under the transcriptional control of their cognate promoter. Cells were restreaked on SC-Leu-Trp plates and then spotted in 10-fold serial dilution steps onto SC-Leu-Trp and SC-Leu-Trp+5-FOA plates, which were incubated for 3 d at 30ºC. **(c)** *In vivo* phenotypes of cells expressing viable Rpl10 variants harboring mutations within the N-terminal residues. YCplac111-based plasmids harbouring the *RPL10* wild-type gene or the indicated *rpl10* alleles, under the control of the cognate promoter, were transformed into the *RPL10* shuffle strain. After plasmid shuffling on 5-FOA-containing plates, cells were restreaked on YPD plates and then spotted in 10-fold serial dilution steps onto YPD plates, which were incubated for the indicated times at 30ºC, 23ºC, 37ºC and 16ºC.

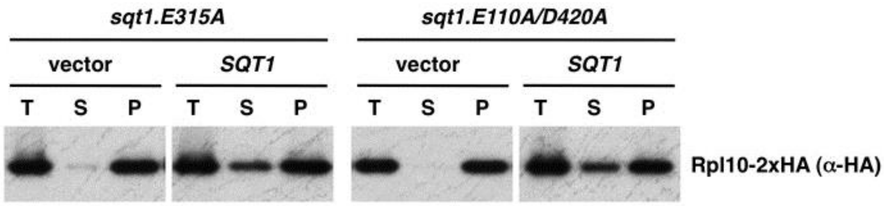

Supplementary Figure 9 Sqt1 is required for the soluble expression of Rpl10 in yeast. Expression of C-terminally 2xHA-tagged Rpl10 (Rpl10-2xHA) was induced for 20 min from the *CUP1* promoter with 500 µM copper sulfate in *sqt1.E315A* or *sqt1.E110A/D420A* mutant cells containing empty vector (vector) or a plasmid expressing Sqt1 from the constitutive *ADH1* promoter (*SQT1*). After cell lysis with glass beads, extracts were centrifuged at 200'000 g for 1 h, and equal amounts of the total extracts (T), soluble extracts (S), and pellet fractions (P) were analyzed by SDS-PAGE and Western blotting using an anti-HA antibody.

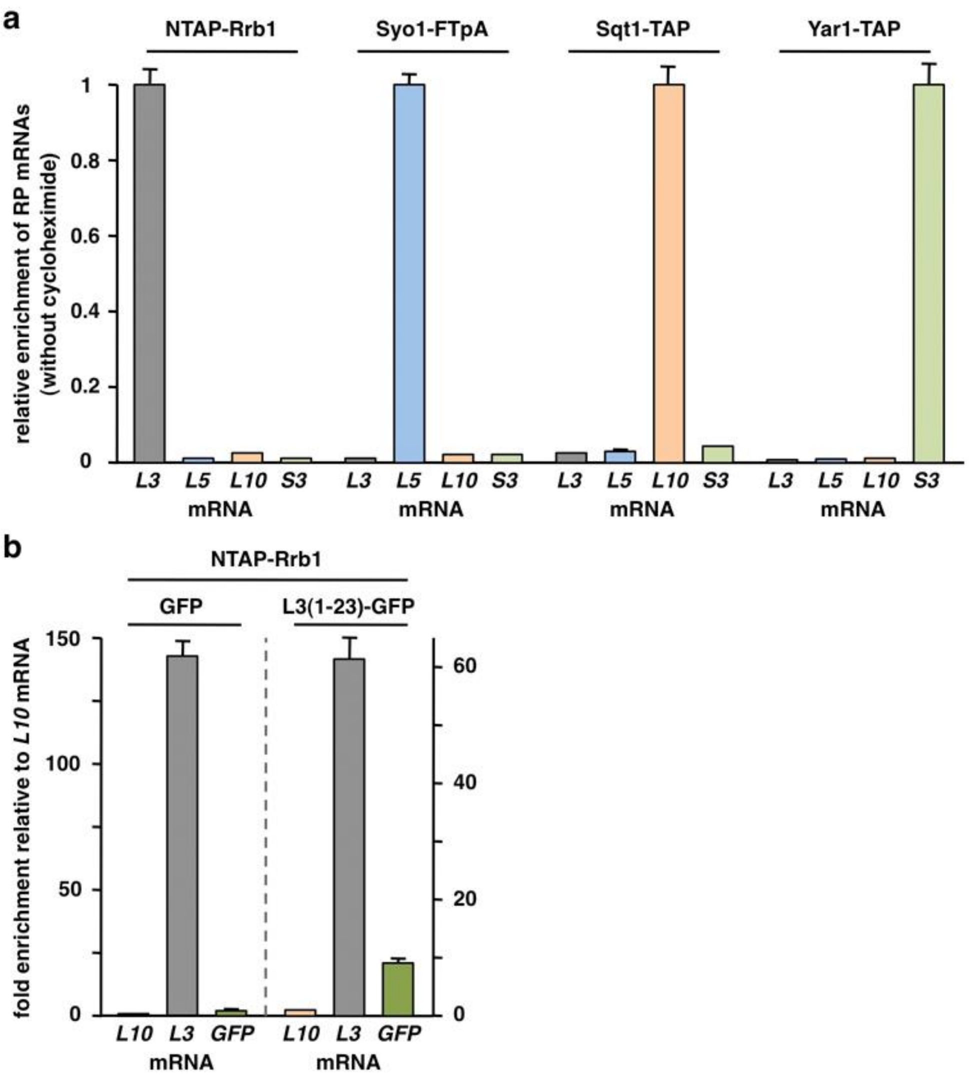

Supplementary Figure 10 Chaperones are recruited to nascent r-proteins. (**a**) The chaperone proteins were affinity purified (IgG-Sepharose pull-down) from extracts of cells that were not treated with cycloheximide and the associated RNA was isolated from the TEV eluates. Each of the four chaperone purifications (NTAP-Rrb1, Syo1-FTpA, Sqt1-TAP, and Yar1-TAP) was assessed for their content of the four r-protein mRNAs (*RPL3*, *RPL5*, *RPL10*, and *RPS3*) by real-time qRT-PCR. The data from one representative experiment are expressed as the relative enrichment of the specifically co-purified r-protein (RP) mRNA in each of the four chaperone purifications (see Methods section for details), with s.d. as error bars. For each cDNA, real-time qPCRs were performed in triplicates. A highly reproducible data set was obtained in an independent series of chaperone purifications. (**b**) The N-terminal residues of Rpl3 are sufficient to target Rrb1 to the nascent Rpl3(1-23)-yEGFP fusion protein. NTAP-Rrb1 was affinity purified (IgG-Sepharose pull-down) from extracts of cells where expression of either the yEGFP (GFP) control protein or the Rpl3(1-23)-yEGFP [L3(1-23)-

GFP] fusion protein has been induced from the *CUP1* promoter for 10 min with 500 µM copper sulfate. Both NTAP-Rrb1 purifications were assessed for their content of the *RPL3*, *RPL10*, and yEGFP (GFP) mRNAs by real-time qRT-PCR. The data from one representative experiment are expressed as the fold enrichment relative to the *RPL10* mRNA, with s.d. as error bars. For each cDNA, real-time qPCRs were performed in triplicates. A highly reproducible data set was obtained in an independent experiment. Note that the bar graphs of the left and right part of this figure are at a different scale.

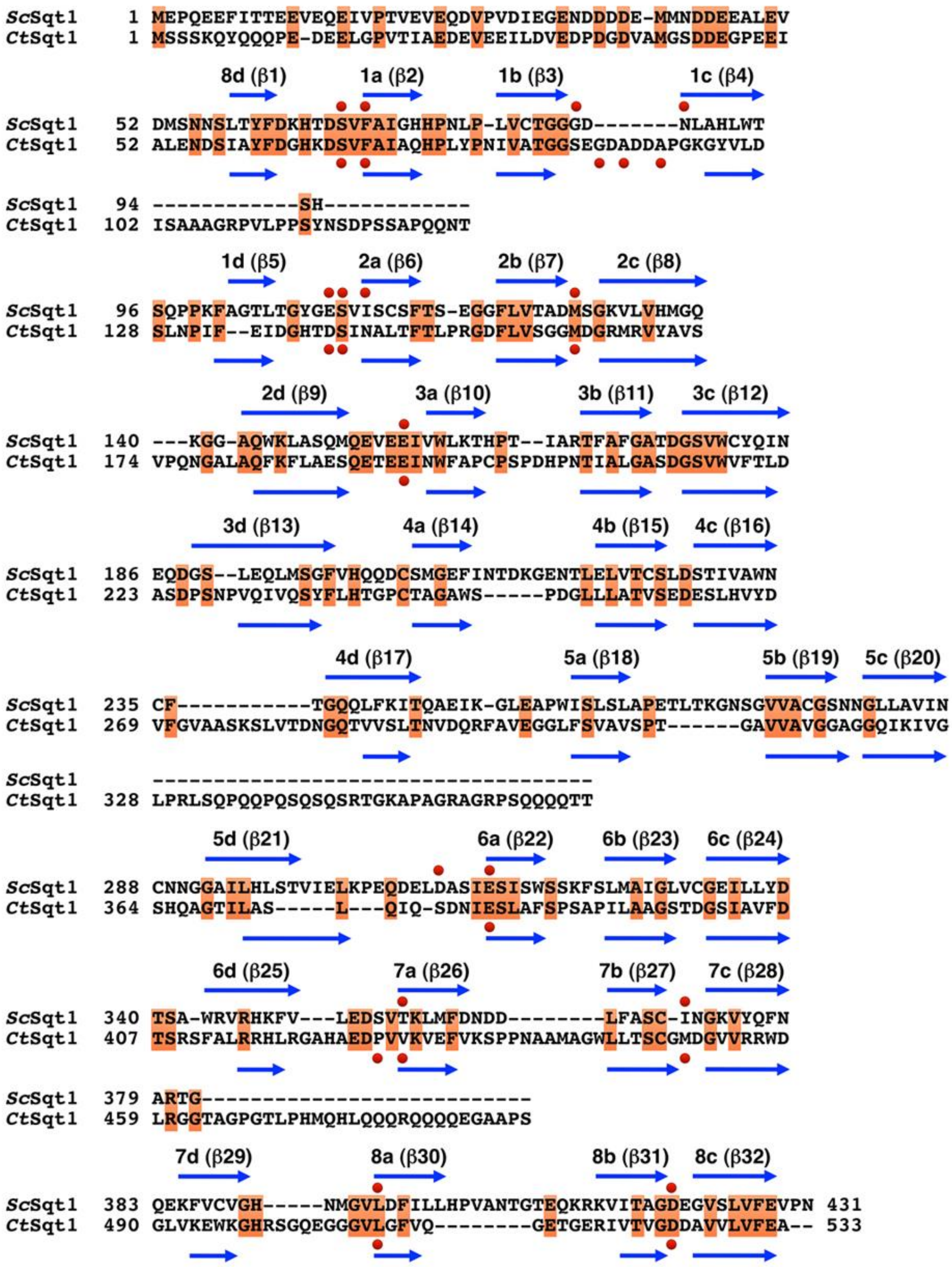

Supplementary Figure 11 Distribution of the Sqt1 residues that contribute to the interaction with Rpl10. The sequence alignment between *Sc*Sqt1 and *Ct*Sqt1 was generated in the ClustalW output format with T-Coffee using the default settings of the EBI website interface[4]. For simplicity, only the identical residues are indicated (orange boxes). Blue

arrows indicate the position of the β-strands (numbered continuously as β1 to β32) according to the crystal structures reported in this study. Assignment of β-strands to the propeller blades (1-8) and labeling of the β-strands within each blade (a to d) is according to the conventional definition for WD-repeat β-propellers. Filled red circles indicate the Sqt1 residues that contribute to the interaction with the N-terminal residues of Rpl10. The binding of the L10-N residues by the top surface of Sqt1 follows the common mode of top-surface-mediated WD-repeat β-propeller interactions with peptides by involving one to two residues per WD repeat that lie at the beginning of the a β-strands (6 out of 8 WD repeats) and mostly one residue per WD repeat at the end of the b β-strands (4 out of 8 WD repeats).

Supplementary Table 1. Yeast strains used in this study.

Name		Relevant genotype	Source
YUS223	*MAT*α	*SQT1*-TAP::HIS3MX4	This study
YUS281	*MAT***a**	*rrb1*::HIS3MX4 *ade3*::kanMX4<br>YCplac111-P*RRB1*-NTAPF-*RRB1*	This study
YUS231	*MAT***a**	*YAR1*-TAP::HIS3MX4	This study
Y4047	*MAT***a**	*SYO1*-FTpA::HIS3MX4	Kressler *et al.* 2012[5]
YUS235	*MAT***a**	*RRB1*-TAP::HIS3MX4	This study
YUS259	*MAT***a**	*sqt1*::natNT2 *ade3*::kanMX4 pHT4467Δ-*SQT1*	This study
YUS256	*MAT*α	*rrb1*::HIS3MX4 *ade3*::kanMX4 pHT4467Δ-*RRB1*	This study
YKL604	*MAT***a**	*rpl10*::natNT2 *ade3*::kanMX4 pHT4467Δ-*RPL10*	This study
YKL636	*MAT*α	*rpl10*::natNT2 *ade3*::kanMX4 pHT4467Δ-*RPL10*<br>*sqt1*::HIS3MX4 YCplac33-*SQT1*	This study
YDK11-5A	*MAT*α	*ade3*::kanMX4	Kressler *et al.* 1999[6]
PJ69-4A	*MAT***a**	*trp1-901 leu2-3,112 ura3-52 his3-200 gal4*Δ *gal80*Δ<br>*LYS2*::*GAL1-HIS3 GAL2-ADE2 met2*::*GAL7*-lacZ	James *et al.* 1996[7]

Strains used in this study were derived from W303 (*MAT***a**/*MAT*a *ade2-1*/*ade2-1 his3-11,15*/*his3-11,15 leu2-3,112*/*leu2-3,112 trp1-1*/*trp1-1 ura3-1*/*ura3-1 can1-100*/*can1-100*)[8]. For yeast two-hybrid interaction assays, strain PJ69-4A was used[7].

Supplementary Table 2. *S. cerevisiae* plasmids used in this study*.

Name	Relevant information	Source
YCplac111-P*RRB1*-NTAPF-*RRB1*	CEN, *LEU2*, P*RRB1*, T*ADH1* N-terminal protA-TEV-CBP-Flag tag	This study
pHT4467Δ-*RRB1*	CEN6 (instable), *URA3*, *ADE3*, P*RRB1*, T*ADH1*	This study
YCplac111-*RRB1*	CEN, *LEU2*, P*RRB1*, T*ADH1*	This study
pGAL111-*RRB1*	CEN, *LEU2*, P*GAL1-10*, T*ADH1*	This study
pADH111-*RRB1*	CEN, *LEU2*, P*ADH1*, T*ADH1*	This study
pG4ADHAN111-*RRB1*	CEN, *LEU2*, P*ADH1*, T*ADH1*, N-terminal G4AD	This study
pHT4467Δ-*SQT1*	CEN6 (instable), *URA3*, *ADE3*, P*SQT1*, T*ADH1*	This study
YCplac33-*SQT1*-stop	CEN, *URA3*, P*SQT1*, T*ADH1*	This study
YCplac111-*SQT1*-stop	CEN, *LEU2*, P*SQT1*, T*ADH1*	This study
YCplac111-*SQT1*	CEN, *LEU2*, P*SQT1*, T*ADH1*	This study
pGAL22-*SQT1*-stop	CEN, *TRP1*, P*GAL1-10*, T*ADH1*	This study
pADH33-*SQT1*	CEN, *URA3*, P*ADH1*, T*ADH1*	This study
pTAPC181-*SQT1*	2μ, *LEU2*, P*SQT1*, T*CYC1*, C-terminal TAP tag	This study
pGAG4ADC111-*SQT1*	CEN, *LEU2*, P*ADH1*, T*ADH1*, C-terminal $(GA)_5$-G4AD	This study
pADH111-*ctSQT1*	CEN, *LEU2*, P*ADH1*, T*ADH1*	This study
pCUP22-*RPL3*-2xHA	CEN, *TRP1*, P*CUP1*, T*ADH1*, C-terminal 2xHA tag	This study
YCplac22-P*RPL3*-yEGFP	CEN, *TRP1*, P*RPL3*, T*ADH1*, C-terminal $(GA)_5$-yEGFP	This study
pyEGFPGAC22-*RPL3*(1-23)	CEN, *TRP1*, P*RPL3*, T*ADH1*, C-terminal $(GA)_5$-yEGFP	This study
pCUP22-$(GA)_5$-yEGFP	CEN, *TRP1*, P*CUP1*, T*ADH1*, C-terminal $(GA)_5$-yEGFP	This study
pCUP22-*RPL3*(1-23)-$(GA)_5$-yEGFP	CEN, *TRP1*, P*CUP1*, T*ADH1*, C-terminal $(GA)_5$-yEGFP	This study
pGAG4BDC22-*RPL3*	CEN, *TRP1*, P*ADH1*, T*ADH1*, C-terminal $(GA)_5$-G4BD	This study
pHT4467Δ-*RPL10*	CEN6 (instable), *URA3*, *ADE3*, P*RPL10*, T*ADH1*	This study
YCplac111-*RPL10*	CEN, *LEU2*, P*RPL10*, T*ADH1*	This study
YCplac22-*RPL10*	CEN, *TRP1*, P*RPL10*, T*ADH1*	This study
YCplac112-*RPL10*	2μ, *TRP1*, P*RPL10*, T*ADH1*	This study
pCUP111-*RPL10*-2xHA	CEN, *LEU2*, P*CUP1*, T*ADH1*, C-terminal 2xHA tag	This study
YCplac111-P*RPL10*-yEGFP	CEN, *LEU2*, P*RPL10*, T*ADH1*, C-terminal $(GA)_5$-yEGFP	This study
pyEGFPC111-*RPL10*(1-20)	CEN, *LEU2*, P*RPL10*, T*ADH1*, C-terminal $(GA)_5$-yEGFP	This study
pCUP22-*RPL10*(1-20)-$(GA)_5$-yEGFP	CEN, *TRP1*, P*CUP1*, T*ADH1*, C-terminal $(GA)_5$-yEGFP	This study
pCUP111-$(GA)_5$-yEGFP	CEN, *LEU2*, P*CUP1*, T*ADH1*, C-terminal $(GA)_5$-yEGFP	This study
pCUP111-*RPL10*(1-20)-$(GA)_5$-yEGFP	CEN, *LEU2*, P*CUP1*, T*ADH1*, C-terminal $(GA)_5$-yEGFP	This study
pGAG4BDC22-*RPL10*	CEN, *TRP1*, P*ADH1*, T*ADH1*, C-terminal $(GA)_5$-G4BD	This study
pFA6a-HIS3MX4	for genomic deletion disruption	Longtine 1998[9]
pFA6a-natNT2	for genomic deletion disruption	Janke 2004[10]
pFA6a-TAP-HIS3MX4	TAP, T*ADH1*; for genomic C-terminal tagging	This study

*For simplicity, only the plasmids containing the respective wild-type genes are listed. The mutant variants thereof used in this study were cloned into the listed plasmids. P and T denote promoter and terminator, respectively.

Supplementary Table 3. *E. coli* expression plasmids used in this study*.

Name	Relevant information	Source
pETDuet-1/*RPL10*-$(His)_6$--*SQT1*-Flag	amp^r, T7 promoter/*lac* operator	This study
pET-15b/*SQT1.53C*-$(His)_6$	amp^r, T7 promoter/*lac* operator	This study
pETDuet-1/*RPL10(1-20)*-$(His)_6$--*SQT1.53C*	amp^r, T7 promoter/*lac* operator	This study
pETDuet-1/*ctRPL10*-$(His)_6$--*ctSQT1*	amp^r, T7 promoter/*lac* operator	This study
pET-15b/*ctSQT1*-$(His)_6$	amp^r, T7 promoter/*lac* operator	This study
pET-15b/*ctSQT1.52C*-$(His)_6$	amp^r, T7 promoter/*lac* operator	This study
pETDuet-1/*ctRPL10(1-20)*-$(His)_6$--*ctSQT1.52C*	amp^r, T7 promoter/*lac* operator	This study

*For simplicity, only the plasmids containing the respective wild-type genes are listed. The mutant variants thereof used in this study were cloned into the listed plasmids.

Supplementary References

1. Ben-Shem, A. *et al.* The structure of the eukaryotic ribosome at 3.0 Å resolution. *Science* **334**, 1524-1529 (2011).
2. Jones, D. T. Protein secondary structure prediction based on position-specific scoring matrices. *J. Mol. Biol.* **292**, 195-202 (1999).
3. Wang, Y., Jiang, F., Zhuo, Z., Wu, X. H. & Wu, Y. D. A method for WD40 repeat detection and secondary structure prediction. *PLoS One* **8**, e65705 (2013).
4. Notredame, C., Higgins, D. G. & Heringa, J. T-Coffee: A novel method for fast and accurate multiple sequence alignment. *J. Mol. Biol.* **302**, 205-217 (2000).
5. Kressler, D. *et al.* Synchronizing nuclear import of ribosomal proteins with ribosome assembly. *Science* **338**, 666-671 (2012).
6. Kressler, D., Doère, M., Rojo, M. & Linder, P. Synthetic lethality with conditional dbp6 alleles identifies Rsa1p, a nucleoplasmic protein involved in the assembly of 60S ribosomal subunits. *Mol. Cell. Biol.* **19**, 8633-8645 (1999).
7. James, P., Halladay, J. & Craig, E. A. Genomic libraries and a host strain designed for highly efficient two-hybrid selection in yeast. *Genetics* **144**, 1425-1436 (1996).
8. Thomas, B. J. & Rothstein, R. Elevated recombination rates in transcriptionally active DNA. *Cell* **56**, 619-630 (1989).
9. Longtine, M. S. *et al.* Additional modules for versatile and economical PCR-based gene deletion and modification in *Saccharomyces cerevisiae*. *Yeast* **14**, 953-961 (1998).
10. Janke, C. *et al.* A versatile toolbox for PCR-based tagging of yeast genes: new fluorescent proteins, more markers and promoter substitution cassettes. *Yeast* **21**, 947-962 (2004).
